# Using an Internet of Behaviours to Study How Air Pollution Can Affect People’s Activities of Daily Living: A Case Study of Beijing, China

**DOI:** 10.3390/s21165569

**Published:** 2021-08-18

**Authors:** Guangyuan Zhang, Stefan Poslad, Xiaoping Rui, Guangxia Yu, Yonglei Fan, Xianfeng Song, Runkui Li

**Affiliations:** 1 IoT Laboratory, School of Electronic Engineering and Computer Science, Queen Mary University of London, London E1 4NS, UK; guangyuan.zhang@qmul.ac.uk (G.Z.); stefan.poslad@qmul.ac.uk (S.P.); guangxia.yu@qmul.ac.uk (G.Y.); yonglei.fan@qmul.ac.uk (Y.F.); 2 School of Earth Sciences and Engineering, Hohai University, Nanjing 211000, China; 3 College of Resources and Environment, University of Chinese Academy of Sciences, Beijing 100049, China; xfsong@ucas.ac.cn (X.S.); lirk@ucas.edu.cn (R.L.); 4 State Key Laboratory of Resources and Environmental Information System, Institute of Geographic Sciences and Natural Resources Research, Chinese Academy of Sciences, Beijing 100101, China

**Keywords:** internet of things, internet of behaviours, air pollution, air quality index (AQI), PM_2.5_, people’s activities of daily living

## Abstract

This study aims to quantitatively model rather than to presuppose whether or not air pollution in Beijing (China) affects people’s activities of daily living (ADLs) based on an Internet of Behaviours (IoB), in which IoT sensor data can signal environmental events that can change human behaviour on mass. Peoples’ density distribution computed by call detail records (CDRs) and air quality data are used to build a fixed effect model (FEM) to analyse the influence of air pollution on four types of ADLs. The following four effects are discovered: Air pollution negatively impacts people going sightseeing in the afternoon; has a positive impact on people staying-in, in the morning and the middle of the day. Air pollution lowers people’s desire to go to restaurants for lunch, but far less so in the evening. As air quality worsens, people tend to decrease their walking and cycling and tend to travel more by bus or subway. We also find a monotonically decreasing nonlinear relationship between air quality index and the average CDR-based distance for each person of two citizen groups that go walking or cycling. Our key and novel contributions are that we first define IoB as a ubiquitous concept. Based on this, we propose a methodology to better understand the link between bad air pollution events and citizens’ activities of daily life. We applied this methodology in the first comprehensive study that provides quantitative evidence of the actual effect, not the presumed effect, that air pollution can significantly affect a wide range of citizens’ activities of daily living.

## 1. Introduction

Continuing human urbanisation exacerbates various physical world conditions, e.g., causing air pollution, traffic congestion, habitat destruction, and loss of arable land [1,2,3]. This threatens the sustainable development of urbanisation by governments [4]. Citizens perceive such negative impacts and are becoming more active to counteract these effects [5]. High levels of air pollution, which can be reflected by a high air quality index (AQI) or PM_2.5_ (air-borne particulate matter having a diameter of fewer than 2.5 micrometres) concentration, have been shown to impact citizens’ health [6], labour productivity [7], later-life educational outcomes [8] and happiness [9]. As mitigation measures, people may choose to live in less polluted cities and in green buildings [10], to use air filtration systems and reduce the time spent outdoors on highly polluted days [11], or decide to wear a breathing mask outside. Governments and businesses may also benefit from understanding the quantitative influence of air pollution on peoples’ activities, such as how much their citizens’ curtail their outdoor activities to avoid bad air pollution.

Although there are some derivative concepts of the internet of things (IoT) that have been applied in many research areas such as the internet of vehicles, a clear definition of the internet of behaviours (IoB) is lacking so we define it as follows. An internet of behaviours (IoB) is defined as a system of IoT devices that collect, use and analyse data about physical (and cyber) human behaviour that seeks to influence human behaviour, i.e., through being better informed about environmental events, and to even trigger changes in human behaviour, en mass, in time and space. One key type of human behaviour critical to human well-being is defined as the basic activities of daily living or ADL, e.g., personal care, mobility, and eating [12]. An IoB system can combine data from multiple IoT environmental sensor sources with commercial customer data, citizen-driven data, data processed by public departments and government agencies, social media, and geographic information science (GIS) data. Based on such data sets, data mining and machine learning enable people’s behaviour to be analysed, then an IoB can enable different stakeholders, e.g., businesses, authorities, citizens to better interpret human behaviour en mass.

To do this, we first need to identify suitable IoB data sources that can be used to track mass human behaviour, such as human movement. For example, call detail records (CDRs) are produced in a cellular phone network base station to document a call, text message, etc., and describe the time of the call, closest base station and location information [13]. Mobile users’ density distribution can be regarded as a high-sampling-rate people’s density distribution that can be computed in time.

It’s useful to differentiate people’s habitual points of interest (POIs) associated with ADLs, such as visiting a favourite restaurant near work. We classify this as a specific place with a specific activity (SPSA), where a location-driven ADL (LD-ADL) occurs, e.g., eating out. These will be differentiated from other POIs where less identifiable LD-ADLs occur (non-SPSA class).

Next, we need to investigate how environmental changes, e.g., air quality affect humans’ behaviour at SPSAs. However, very few studies have quantitatively assessed the impact of air pollution on ADLs, especially at a large, city-wide scale. The main reasons for this are: first, some ADLs are composite and difficult to describe with the data acquired. Second, datasets that can be used to calculate ADLs in a large area, at a sufficiently high sampling rate, can be difficult to obtain unless one works for large telecoms, internet app or social networking companies.

To analyse how people’s ADLs are affected by bad air pollution, we used high-frequency peoples’ density distributions calculated using CDR data, and air pollution data, across the whole of Beijing, China in February 2015, acquired from a telecom provider (China Mobile). According to the national time use survey bulletin of China in 2018 (2015 is relatively close to 2018), ADLs in China have changed little from the previous survey of China in 2008 as conducted by the National Bureau of Statistics of China (http://www.stats.gov.cn/tjsj/zxfb/201901/t20190125_1646796.html, accessed on 28 August 2020).

In Table 1, the time spent on SPSA ADLs including sleeping, eating, housework, fitness exercise, watching TV, and transportation, account for more than 60% (=63.74%) of a day.

Hence, we select four representative types of SPSA ADLs: sightseeing (N.B. sightseeing has been shown to have a strong effect on human well-being [14]), eating out, staying-in (to rest and recuperate) and the type of transport mode used. Each type of SPSA ADL is associated with a point of interest (POI) dataset of fixed places, except for the transport mode used. Some transport modes, e.g., bus and subway can be detected by fixed POIs, while others could be extracted from the movement speed. However, people who take taxis or cars can be hard to recognize as they have no fixed POIs and can have a similar speed to buses and cycles, during traffic congestion. According to the Beijing Traffic Development Annual Report in 2016 (http://www.bjtrc.org.cn/, accessed on 1 August 2019), the total average travel distance travelled using bus, subway, cycling, and walking is 26.1 km, which accounts for 53% of the total average distance (49.2 km). Their time duration accounts for 65% of the total average travel time (485 min). Thus, we focus on four types of transport modes: bus, subway, cycling and walking.

Our key and novel contributions are as follows:

(1) We propose a methodology to better understand the link between environmental changes such as air pollution and citizens’ activities of daily life. It can help government and businesses to understand better the actual effect not the presumed effect of air pollution on the pattern of daily activities of citizens;
(2) This opens up a new perspective for understanding and exploring the interaction between PM_2.5_ and in more general air pollution and people’s physical behaviour.
(3) This can not only reveal the subtle impact of PM_2.5_/air pollution on human ADL but can also monitor the indirect impact of PM_2.5_/air pollution on some human-based business activities, e.g., restaurants. This is challenging to do because different data sets, such as air pollution, human movement, location contexts, etc., with different temporal and spatial characteristics, need to be acquired and fused. Human activities can be complex to characterise. Individual human behaviour in a crowd needs to be identified. Human behaviour is affected by a range of environmental factors, some of which may not be observable, that need to be correlated.

The remainder of this article is organized as follows: Section 2 analyses related work, Section 3 presents the data and pre-processing used. Section 4 introduces an overview of the methodology, followed by a more detailed description. Results and discussion are reported in Section 5. Section 6 gives our conclusions, limitations, and thoughts for future research. The abbreviations are explained in Table 2.

## 2. Related Works

Several factors affect crowd activities, including human factors (such as leaving work) and physical environment factors (such as temperature, and rain). Bad air quality can affect the outdoor activities of residents [15]. To study how the influence of haze (air pollution with high degrees of PM) affects different crowd activities in different urban areas, we first consider regression models that establish regression relationships between weather factors and specific human behaviour. De Freitas [16] observed that atmospheric conditions affect beach user behaviour. Lin et al. [17] found that poor air quality causes the elderly to stay indoors. Jiang et al. [18] used a social media survey and regression and variance analysis to find that particulate pollution negatively impacts the maximum number of park visits. R-Toubes et al. [19] analysed the relationship between weather conditions and people flow, daily, at tourist beaches highlighting that sunshine is important. Zhao et al. [20] found via a survey that in hazy weather, higher-income cyclists in Beijing tended to switch to use private vehicles rather than to use public transit, while lower-income cyclists were more likely to continue cycling. Hu et al. [21] used a multivariate regression method to study the relationship between air quality and outdoor exercise in China. The total number of exercise sessions, average duration and an average distance of each exercise mode, were analysed under each air quality category (from excellent to severe).

Omitted variable bias is a primary statistical challenge in nonexperimental research (research that lacks the manipulation of an independent variable, control of extraneous variables through random assignment, or both). Fixed effect models (FEMs) with panel data were developed to address the issue of omitted variable bias in nonexperimental research [22]. Thus, FEMs can be applied to detect the relationship between ADLs and air pollution where the model is an estimation technique, employed on panel data that allows one to account for time-invariant unobserved individual characteristics, i.e., other factors such as special offers at different times of day, etc., that can be correlated with the observed independent variables (AQI or PM_2.5_) [23]. For example, Gao et al. [24] studied the impact of different air pollutants on dining-out activities and the satisfaction of urban and suburban residents. They found that due to differences in environmental and health awareness, the impact of air pollution on dining-out behaviours varies among urban and suburban residents. Zheng et al. [25] studied how air pollution affects residents’ eating out frequency and satisfaction based on the reviews from dianping.com. They proposed that air pollution can reduce the dining-out frequency and satisfaction of residents. However, in both studies, they collected residents’ dining-out data from only one third-party website. This is not subjective because not everyone tends to leave a review on the website. In contrast, CDR data is much more representative because people’s locations are recorded and computed with a higher frequency and the data is objective. Further, when using a FEM, both two studies did not take measures to solve the potential endogeneity issue brought about by an omitted variable [26], which leads their results to lack robustness. Table 3 summarizes the above related work along with their ADL, data, method, and limitations.

In conclusion, current studies of how air pollution can affect physical human behaviour have the following specific limitations: (1) Current studies only focus on one, or very few, type(s) of ADLs; (2) they do not clarify the differences between haze, AQI, and PM_2.5_; (3) they do not study how to model the link between air quality and multiple types of ADLs at a large spatial scale and (4), although they consider other observable impact factors that may influence the ADLs using traditional questionnaires survey or simple statistic regression models, there are many other unobservable or unquantified factors that may also have a significant influence that are not considered. Even though (4) could be solved by using a FEM, current FEM air quality studies still have problems of (5) data objectivity and (6) model robustness. Thus, in this study, our contributions also include the solutions to solve the six limitations mentioned above.

## 3. Data and Pre-Processing

### 3.1. Data Introduction

Here, the main datasets CDR and air pollution and any preprocessing are introduced. Other datasets, weather conditions, POI and building area are introduced in Appendix A.4. It is challenging to identify and fuse such heterogeneous data about POIs that may have different temporal and spatial characteristics such as resolution.

#### 3.1.1. Call Detail Record (CDR)

**Figure 1:**
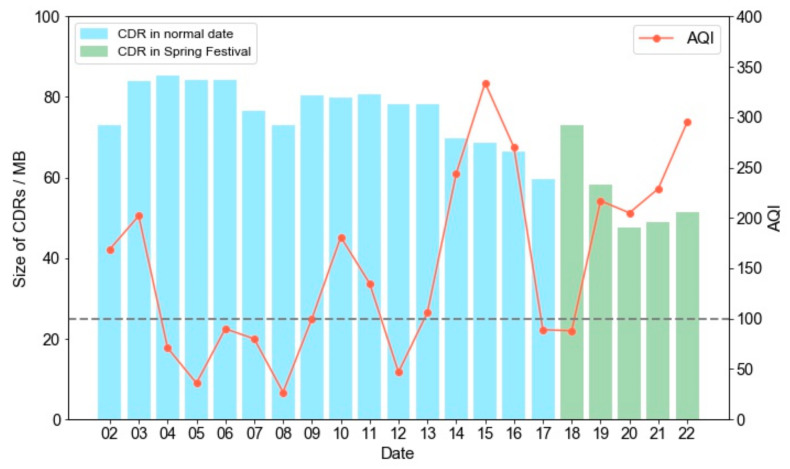
Daily CDR files’ sizes and AQI over the study period (N.B. the left y-axis represents the data size in megabytes, the x-axis date represents a day in the month in February 2015. The dotted line is a threshold AQI of 100 and represents a poor AQ in which it’s recommended that sensitive citizen groups should cut back or reschedule strenuous outdoor activities).

People’s activities can be reflected by how their distribution density changes when they visit different POIs derived from their anonymised individual mobile phone CDRs via a base station positioning method [27] because of the high penetration rate of smart mobile phones according to the World Telecommunication Development Conference (2014). CDRs from a telecommunication operator with the highest customer number in China from Monday 2 February to Sunday 22 February 2015 (21 days) were analysed. This dataset includes over 4.8 billion records for more than 300 million users per day. The size of an hourly CDR file is about 2 Gigabytes (Figure 1). The CDR processing details are reported in Appendix A.1, Appendix A.2, Appendix A.3.

Figure 1 shows how the CDR file size distribution varies with AQI over the study period. In major national holiday periods such as the Spring Festival in China (from 18 February 2015), there is a considerable fluctuation in file size because of a significant people movement, which makes this research close to a natural experiment [28]. Additionally, because of the reasons introduced in Section 3.2 and Appendix A.4.1, the focus here is on the analysis of the relationship between people’s activity and air quality from sunrise to sunset and the analysis of the corresponding relationship at nighttime is omitted.

#### 3.1.2. Air Pollution

China’s Ministry of Environmental Protection (MEP) reports real-time hourly concentrations for the major air pollutants such as PM_2.5_, PM_10_ (particulate matter with an aerodynamic equivalent diameter of less than 2.5 and 10 μm, respectively), SO_2_, O_3_, NO_2_ and CO at about 1000 monitoring stations. Beijing has 35 of these.

Among these six pollutants, MEP defines a city’s “primary pollutant” as the pollutant which contributes the most to the air quality degradation on an hourly basis. MEP also releases a composite air quality measure, AQI, which is calculated hourly.

PM_2.5_ seems to dominate more in recent years [29]. Its smaller size makes PM_2.5_ much more harmful for people’s health than larger particulates, such as PM_10_. Baidu.com, China’s most popular online search engine. which accounts for 93% of the search engine penetration rate in 2015 (CNNIC, 2015) and 91% in 2019 (CNNIC, 2019), shows that Chinese citizens’ concerns for PM_2.5_ are about 12 to 500 times higher than those of other air pollutants in the past several years (as computed using the Baidu Search Index tool http://zhishu.baidu.com/v2/index.html#/, (accessed on 1 March 2021). Thus, we use both PM_2.5_ and AQI to detect their relationship with people’s activity in our study. Other key pollutants (PM_10_, SO_2_, O_3_, NO_2_, CO) have been considered and are reported in Appendix A.4.1.

### 3.2. Data Collection and Accuracy Analysis

Excluding the CDR data, the data used in this study is measured by scientific calibrated instruments managed by national air pollution stations and collected according to international standards. The details of the sensors used to collect the data, including their measurement range, resolution, and accuracy, are reported in Table 4.

Due to the different measuring principles of multi-sensors, the definitions of the accuracy of these differ. For the p.m. data, the accuracy could be reflected by parallelism of monitors (PoM), effective data rate (EDR) and comparison test of reference method (CTRM). In Table 4 we only show the EDR of the p.m. (others are reported in Appendix A.4). For other pollutants, the accuracy is defined as the indication error, while for weather condition data, the maximum allowable error could reflect the accuracy.

Although the data collection processing should meet the request of the international specifications, which ensures that the accuracy and repeatability of the datasets have qualified with the specifications before they were published as an open-source, sensors may fail which leads to inaccuracies in the data. Thus, we design our method set up as a randomly sampled experiment which serves as a cross-check of our results. This processing can eliminate the resulting error caused by sensor failure and other factors.

For the CDR data, we have computed the spatial accuracy to be about 500 m when estimating mobile phone users’ density distributions, which has a high spatial resolution to recognise the ADLs of people based on their location. More details can be found in Appendix A.2, Appendix A.3, Appendix A.4

### 3.3. Data Pre-Processing

The CDR dataset is converted into hourly dynamic mobile phone users’ density distributions to then extract people’s dynamic activity at specific POIs. First, we extract the first 5 min of CDRs from each hour file as a representative sample of each hourly CDRs (to reduce the computation time) and then count the unique International Mobile Subscriber Identification Number (IMSI) as a representative sample mobile phone user. From the CDRs, we can derive the hourly density distribution of mobile phones corresponding to each VP (Voronoi polygon or cell—the area representing the coverage) of the base station. However, an unknown error may be caused by the uneven distribution of VPs, leading to an uneven positioning accuracy resolution, even though the POIs are evenly spatially sampled, e.g., the people density for a POI within a small VP is more accurate than for a POI in a big VP. To alleviate this, we use the kernel density estimation (KDE) method [30] to estimate hourly density distributions for a city (see Appendix A.3), with the raster resolution parameter set as 500 m (as justified and reported in Appendix A.2)

Through using an inverse distance weighting (IDW) method [31] to interpolate the point values of air quality from 35 monitor stations in a city (Beijing), we map the hourly dynamic AQI and PM_2.5_ density distributions and build the corresponding two spatial-temporal datasets. Then, we can extract the AQI and PM_2.5_ values for specific POIs, at a density that is similar to the CDR-based density.

## 4. Methodology

### 4.1. Overview

**Figure 2:**
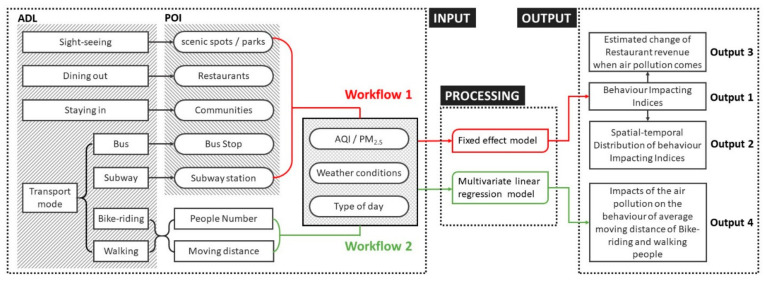
Overview of the methodology for the IoB framework.

Two workflows are defined for our IoB framework (see Figure 2) based on if an ADL uses a POI that acts as an SPSA or not. Both workflows include three modules: input, processing, and output.

Workflow 1 (red parts in Figure 2) focuses on the first category of ADLs, which covers four ADLs represented by five fixed POIs that are SPSAs: (1) sightseeing, (2) eating out (restaurants are POIs), (3) staying-in, (4) travelling by bus (a transport mode that uses bus stops as the POIs), and (5) travelling by subway (that uses metro stations as the POIs). The inputs include the mobile phone users’ density distributions for these five fixed POIs and the corresponding air pollution, weather conditions and types of day datasets. Next, the processing module is used to build FEMs to detect the relationship between people’s ADLs and air pollution, where any behaviour impacting indices (a coefficient in a FEM for people’s density, e.g., β in Equation (1)), are computed as the outputs (Output 1). Based on the overall POI distributions in the whole city, the spatial-temporal distribution of the behaviour impacting indices is mapped (defined as Output 2).

In addition, before using CDRs to extract peoples’ density values, we analyse if the spatial resolution of the calculated people distribution is accurate enough to extract values to represent the four ADLs (see Appendix A.2). We conclude that except for the restaurant POI/ SPSA ADL, all other four kinds of POIs can be used to represent the related SPSA ADLs in Strategy 1 (S1). Thus, to decrease the estimation error when processing FEMs for the restaurant POI, we present Strategy 2 (S2) that samples Voronoi polygons (VP) that includes large areas of restaurants (Appendix B). Then, S2 can also be applied to Workflow 1. Finally, Output 3 estimates changes in restaurant revenue due to air pollution based on the behaviour impacting indices.

Workflow 2 (green parts in Figure 2) focuses on the second category of non-SPSA ADLs, which covers one ADL, represented by the two transport mode POIs: (1) walking, and (2) cycling. For these inputs, a multivariate linear regression model is used to compute how many people tend to cycle or walk and whether they would curtail the distance travelled or change transport modes. Output 4 indicates the quantitative impact of air pollution on the average moving distance of people walking and cycling.

Note that the testbed to build the FEM and multivariate linear regression model is STATA16 (https://www.stata.com/, accessed on 7 August 2021), while the GIS related data is processed in ArcGIS 10.5 (https://www.esri.com/en-us/arcgis/about-arcgis/overview, accessed on 7 August 2021). Further, in terms of Workflow 1, we conduct random experiments to extract half of the POIs in each dataset randomly 10 times, and then the effect of air pollution on four ADLs influenced by significant changes of the AQI and PM_2.5_ is investigated (Table A7, Table A8, Table A9, Table A10 and Table A11). Then after getting the results of the random experiments, we build FEMs for all POIs (SPSAs) and the results are reported in Table A12, Table A13, Table A14, Table A15 and Table A16. Furthermore, the random sampled experimental settings could be regarded as validation processing. The strategy of the validation can solve the potential endogeneity issue brought about by an omitted variable [26], as well as eliminate the error from inaccurate sensor data. Based on the 10 experimental results, we determine that PM_2.5_ as part of an AQI influences people’s activity only when the percent of significant coefficient (*p*-value < 0.05) is more than 60%.

### 4.2. Workflow 1

#### 4.2.1. Input Module: Data Preparation and Input Set Up

**Figure A5:**
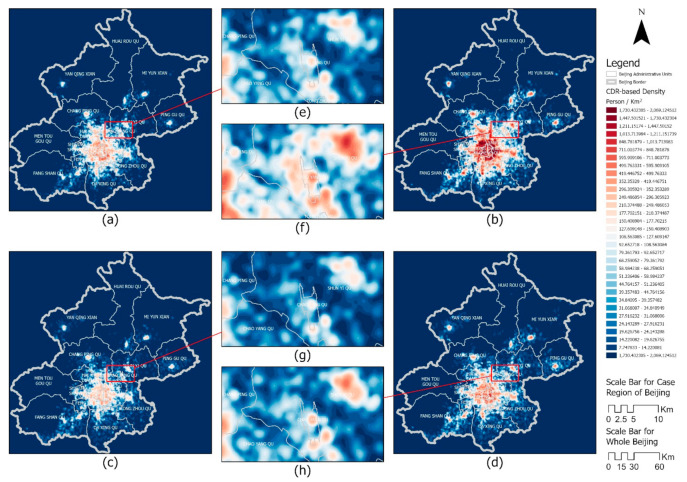
Four examples of the CDR-based people density distribution at different time points. (**a**–**d**) show the distributions in the whole of Beijing, while (**e**–**h**) illustrate an inner area of Beijing to show more details. (**a**,**e**) are at 6:00 a.m. 2 February 2015 while (**b**,**f**) are at 6:00 p.m. on the same day. (**c**,**g**) are at 6:00 a.m. 19 February 2015 (Spring Festival), while (**d**,**h**) are at 6:00 p.m. on the same day.

At nighttime people tend to sleep and communicate less and interact less with the base station so these CDRs do not reflect the real users’ density distribution during such a study period. Hence, we focus on the daytime period from 6:00 a.m. to 7:00 p.m. (13 h) when studying mobile phone users’ records and also because in this period people can visually perceive the main air pollution. Some examples of the spatial-temporal distribution of the CDR-based people density are given in Figure A5 which is discussed later in Section 5.1. We merge the mobile phone user’s density data with the POI-level hourly AQI, PM_2.5_ concentration data and weather data. Data sources, definitions and summary statistics of the main variables are provided in Table 5.

We divide each day into different time slices for different types of ADLs. For sightseeing, using different transport modes, and staying-in, we define three daily periods, from 6 to 10 a.m. (P1), from 10 a.m. to 2 p.m. (P2) and from 2 to 6 p.m. (P3). So that the three periods each span 4 h, hence, the time slot 6 p.m. to 7 p.m. is excluded. For the eating out ADL, we consider two periods in each day: the lunch period 11 a.m. to 2 p.m. (P-Lunch) and dinner period 5 to 8 p.m. (P-Dinner). We omit the influence when sunset happens after 6 p.m. for eating out. For every period, we calculate the mean value of the POI-level hourly mobile phone user density, AQI, PM_2.5_ concentration data and weather data, and then analyse these.

#### 4.2.2. Processing Module, Output 1: Fixed Effect Model (FEM)

To study the main effect of air pollution on people’s activity, we use a FEM panel regression approach as shown in Equation (1):

(1) $$\begin{array}{c} Y_{it}=\alpha_{0}+\alpha_{i}+\beta X_{it}^{’}+\delta Z_{i}^{’}+\varepsilon_{it}\\ =u_{i}+\beta X_{it}^{’}+\delta U_{i}+\varepsilon_{it},\;i=1,2,\ldots ,N,t=1,2,\ldots ,T \end{array}$$

(2) $$X_{it}={\left(X_{1it},X_{2it},\ldots ,X_{kit}\right)}^{’},\beta ={\left(\beta_{1},\beta_{2},\ldots ,\beta_{k}\right)}^{’}$$

Here, $Y_{it}$ is the dependent variable, which changes w.r.t the time and individual. $\alpha_{0}$ is the constant, while $\alpha_{i}$ is the individual effect which is time-invariant. We can set $u_{i}=\alpha_{0}+\alpha_{i},E\left(\alpha_{i}\right)=0,E\left(u_{i}\right)=\alpha_{0}$, where the unobservable random variable $u_{i}$ represents the intercept term of individual heterogeneity, called the individual effect. $X_{it}$ is a k×1 vector representing the independent variables/ β is a k×1 vector representing the correlation coefficients of $X^{\prime }$ (Equation (2)). $Z_{i}^{’}$ is the unobservable independent variable which is time-invariant. $\varepsilon_{it}$ represents the idiosyncratic error. N is the index number of the individual. T represents the time number index.

The FEM method estimates the coefficient of air pollution impacting on people’s ADLs, which is shown as below: First, fixing the i in Equation (1), the time is averaged, giving:

(3) $${\bar{Y}}_{i}=u_{i}+\beta {\bar{X}}_{i}^{’}+\delta Z_{i}^{’}+{\bar{\varepsilon }}_{it}$$

(4) $${\bar{Y}}_{i}\equiv \frac{1}{T}\overset{T}{\underset{t=1}{\sum }}Y_{it}$$

While ${\bar{X}}_{i}^{’}$ and ${\bar{\varepsilon }}_{it}$ have similar definitions. Then, using Equation (1) minus Equation (3) we get:

(5) $$Y_{it}-{\bar{Y}}_{i}=\beta {\left(X_{it}-{\bar{X}}_{i}\right)}^{’}+\left(\varepsilon_{it}-{\bar{\varepsilon }}_{i}\right)$$

In this step, $Z_{i}$ and $u_{i}$ have been eliminated. Then we define ${\tilde{Y}}_{it}\equiv Y_{it}-{\bar{Y}}_{i}$, ${\tilde{X}}_{it}\equiv X_{it}-{\bar{X}}_{i}$, ${\tilde{\varepsilon }}_{it}\equiv \varepsilon_{it}-{\bar{\varepsilon }}_{i}$, we get:

(6) $${\tilde{Y}}_{it}=\beta {\tilde{X}}_{it}+{\tilde{\varepsilon }}_{it}$$

Finally, we use the ordinary least squares (OLS) method to estimate β, which is called the Fixed Effect Estimator, ${\hat{\beta }}_{FE}$.

In our study case, the dependent variable is an ADL, represented by the CDR-based people density for a specific POI. Independent variables are classified into three categories: pollution (AQI/PM_2.5_), weather conditions (temperature, wind speed, cloud cover rate, rainfall, snowfall) and type of day. Because in the study period, there is no rainfall or snowfall, FEM is defined in Equation (7):

(7) $$\begin{array}{c} DENSITY_{it}=u_{i}+\alpha_{1}POLLUTION_{it}+\alpha_{2}TEMP_{t}+\alpha_{3}WIND_{t}+\alpha_{4}CLOUD_{t}+\alpha_{5}TD_{t}+\delta Z_{i}^{’}+\varepsilon_{it},\\ i=1,2,\ldots ,N,t=1,2,\ldots ,T \end{array}$$

$DENSITY_{it}$ and $POLLUTION_{it}$ represent the people density and the pollution level of POI i at time t, respectively. In the FEM, we use AQI and PM_2.5_ concentrations as the pollution variable, respectively. $TEMP_{t}$, $WIND_{t}$, $CLOUD_{t}$ represents temperature, wind speed, cloud cover rate (it only has a t index as all the POIs have the same weather values at the same time). $TD_{t}$ refers to a type of day dummy variable. To control the time-invariant unobservables that vary across cities, we include the POI fixed effect $\delta Z_{i}^{’}$. Note that unobserved factors are not classified, they are known unknowns and just grouped. Coefficient $\alpha_{1}$ (is the corresponding coefficient of ${\hat{\beta }}_{FE}$) reflects people’s pollution responsiveness, which should be negative, while other coefficients $\alpha_{1}$, $\alpha_{2}$, $\alpha_{3}$, $\alpha_{4}$, and $\alpha_{5}$ correspond to other independents observed variables. N is set as the POI number. T is set as 21 (days).

Before using the FEM in our method, the panel unit root test (PURT) is applied to each variable to see if it is unstable or not, which can avoid spurious regressions [32]. We chose the Im-Pesaran-Shin (IPS) test [33] method for this. Then we use the Harris-Tzavalis (HT) method to test for stationarity to see if the statistical properties of the time series change over time [34]:

(8) $$\begin{array}{r} H_{0}:\;\rho_{i}=1\;\\ H_{1}^{IPS}:\;\rho_{i}<1,i=1,2,\ldots ,N_{1};\rho_{i}=1,i\\ \;\;\;\;\;\;\;\;=N_{1}+1,N_{1}+2,\ldots ,N\;\\ \underset{N\to \infty }{\lim}\left(N_{1}/N\right)=H_{0},0<\delta_{1}\leq 1 \end{array}$$

Equation (8) shows the IPS hypotheses, $H_{0}$ and $H_{1}^{IPS}$. If the test result rejects $H_{0}$, that means the tested data is stable. Further for the HT test shown in Equation (9), if the test result rejects $H_{0}$, this means the tested data is stable:

(9) $$H_{0}:\;\rho =1\;H_{1}^{HT}:\;\left|\rho \right|<1$$

The results show that the variables are all stable even though they span the Spring Festival holiday period, which means the condition of our datasets satisfies this requirement and hence, we can effectively use a FEM.

The type of day, such as weekday, weekend day, festival day, may cause a major impact on people’s activity in different periods, we thus represent the influence of the type of the day as a dummy variable in the FEM. Furthermore, in many similar previous studies [9], weather-related conditions also play a key role in the analysis using FEMs.

In the next step, we input weather conditions, such as the temperature value squared to examine the non-linear effect of it on people’s activities following [9]. We give the variable definitions and summary statistics in Table 5. We recognise that the actual relationship between air quality and people’s activity may be generated by omitted variables that represent unknown factors that vary hourly for individual POIs. For example, historical POI sights in Beijing may be visited by tour groups outside Beijing, whose time plan would not likely be changed by bad air quality and even weather conditions as there may be no alternative day to visit such a sight.

#### 4.2.3. Output 2: Spatial-Temporal Distribution of Behaviour Impacting Indices

We create and display a summary of the spatial-temporal distribution of the area impacted by air pollution, city-wide as follows. First, a city is divided into grids of 5 km×5 km cells. Second, five types of POIs for the four ADLs are counted in every cell. Then the corresponding correlation coefficient is used as a weighting factor to create a summary behaviour impacting index and map the 3-dimension distributions for the three different 4-h daily periods. Equation (10) computes the summary behaviour impacting index for every cell as follows:

(10) $$INDEX_{cell}=\overset{m}{\underset{i=1}{\sum }}A_{i}N_{i}$$

where $N_{i}$ is the i-th POI number that represents ADLs in a cell, m is the number of types of POIs, 5 in this study. *A* is the corresponding value of the correlation coefficients (Table A12, Table A13, Table A14, Table A15 and Table A16). The final INDEXs are shown as the different bars for the three periods. The index for each different period of a day is computed independently, so there are three distributions. We use two colours to distinguish the negative and positive final effects of air pollution: red means negative and green means positive.

#### 4.2.4. Output 3: Estimating the Revenue Change

Then we describe how the influence of air pollution on the revenue of restaurants in Beijing is calculated. We use the correlation coefficient to represent the impact of AQI and PM_2.5_ on people who eat out in restaurants during the lunch period. After getting the significant value (*p*-value) from the AQI or PM_2.5_ impact $\alpha_{1}^{r}$ by the FEM, which means that when AQI or PM_2.5_ change by one unit quantity, the density of sampled people reflected by mobile phone users’ density in the restaurant VP decreases by $\alpha_{1}^{r}$ people/km^2^. Then because we use mobile phone users as the sampled people in Beijing, there is a sampling rate SR. Thus, if PM_2.5_ increases by 1 μg/m^3^, the density of people in a restaurant VP would decrease by $\alpha_{1}^{r}\times SR$ people/km^2^. Then the average area of the sampled restaurant A is computed. Based on the per capita consumption (PCC), considering the different cost levels of restaurants is 19 Chinese Yuan (CNY) [35], we can calculate that if the AQI or PM_2.5_ change by one unit quantity. The total number of people who go to the restaurant for lunch would change by $\alpha_{1}^{r}\times SR$ and the change in average revenue (CAR) of all restaurants in Beijing is computed using Equation (11) in one day as follows:

(11) $$CAR=\alpha_{1}^{r}\times SR\times A\times PCC$$

To estimate the total change of the revenue of the restaurant when air pollution comes, the change in air pollution (Set to unknown x unit quantity) is used to estimate the final change in revenue of the restaurant by multiplying x by CAR.

### 4.3. Workflow 2

#### 4.3.1. Input Module: Walking and Cycling People Data

We consider if the other two transport mode groups, cycling and walking, are impacted by air pollution. For each group, two indicators of the responding people’s number and movement distance are calculated hourly during the study period. These constitute six time series. After combining the weather conditions and type of day, we test the autocorrelation for each time series using the Ljung–Box test method [36], drawing the conclusion that all the time series have at least a first-order autocorrelation. Then we input the data into the Transport mode options analysis model as shown above and we use the Prais-Winsten method [37] to estimate the relationship between the activity of the two groups and air quality respectively.

From the CDRs, we can also extract a single user’s trajectory based on a person’s unique identity IMSI. The base station records a user’s IMSI with a timestamp when this is combined with the location of the base station. We can derive the distance between two or more base stations to represent people’s movement and then we can calculate the speed of people moving in a specific period. We define these two features as people’s CDR-based moving distance and CDR-based moving speed. When this speed is within a range, it is believed that the mobile phone user is using a specific transportation mode. The experiments of Wang et al. [38] confirm that this method can generally obtain an 80–90% accuracy when inferring simple transportation modes, e.g., walking and driving. Furthermore, within the same case region, Beijing, Wang et al. [39] utilize CDR data to analyse travel distance between traffic zones and conclude that CDR data use for traffic mode analysis is feasible. Bwambale et al. [40] use the logit model to prove that CDR can capture the expected behaviour towards overlapping routes. All these studies demonstrate that CDR-based trajectories have very similar features to the ground truth ones for distance and speed.

According to [41], the average bike speed was 9.1 km/h in Beijing, and the walking speed was on average 5 km/h [42]. In each hour, the first 5 min is still sampled, and then all unique users are extracted using the unique IMSI. For each user or sample person, we get all the records sorted based upon continuous-time nodes and calculate the distance and speed in each section (defined as when one person moves from one base station to the next base station). If the speed is within 7 to 10.5 km/h in one section, we judge the user as a bike-riding person and then add one to the total number of this group and calculate the total distance in all cycling sections for this person. Walking has a speed lower than 7 km/h. Finally, we sum the number of people and total distance travelled respectively for each group. We get the two-time series datasets for distance and speed.

For the number of people in a group, we can easily calculate this from the CDR data, while we use Equation (12) to calculate the distance travelled by people:

(12) $$DISTANCE_{t}=\overset{n}{\underset{i=0}{\sum }}\overset{m}{\underset{j=0}{\sum }}D_{ti}\left(P_{tij},P_{ti,j+1}\right)$$

where $DISTANCE_{t}$ represents the summary distance of all people who have moved at hour t; n is the number of people who have moved in hour t. i is the identity. $D_{ti}\left(P_{tij},P_{ti,j+1}\right)$ is the final distance of $Person_{i}$ in hour t, calculated using the accumulated length from point j to point $\left(j+1\right)$.

#### 4.3.2. Processing Module: Multivariate Linear Regression Model

After getting the number and distance of each group of people, we calculate the average value of AQI, PM_2.5_ concentration and weather conditions within the whole of Beijing. Then we use a multivariate linear regression model from Equation (13) to estimate the impact of air quality on people’s activity of these two groups respectively. The dependent variable is the number of, or the distance moved by people, which is defined as an ND-features of people. The independent variables include air quality, weather conditions and type of day as follows:

(13) $$\begin{array}{c} ND_{FEATURE}{}_{t}=\beta_{0}+\beta_{1}POLLUTION_{t}+\beta_{2}TEMP_{t}+\beta_{3}WIND_{t}+\beta_{4}CLOUD_{t}+\beta_{5}TD_{t}+\varepsilon_{t},\\ t=1,2,\ldots ,T \end{array}$$

where $ND_{FEATURE}{}_{t}$ represents the ND-features and $POLLUTION_{t}$ (AQI/PM_2.5_), while $\beta_{2}TEMP_{t}$, $\beta_{3}WIND_{t}$, $\beta_{4}CLOUD_{t}$ and $\beta_{5}TD_{t}$ control the weather conditions and type of day effects. $\beta_{1}$ reflects the relationship between the ND-feature and air pollution. Because all variables are time series data, they have the potential for autocorrelation. Thus, we use the Prais-Winsten method [37] to estimate $\beta_{1}$, which aims to decrease the influence of temporal autocorrelation.

#### 4.3.3. Output 4: Average Distance Changing of Walking and Cycling People

$\beta_{1}$ represent a feature unit that changes when POLLUTION changes by one unit. For example, if the results of $\beta_{1}$ are significant, at a 95% confidence level (*p* < 0.05), when AQI changes by one unit, the number of people who cycle changes by $\beta_{1}$ units. If we get the two statistically significant level values $\beta_{1}$ of the number, and the distance of, people walking or riding a bike, the relationship function between POLLUTION and AverageDistance of the specific group can be calculated directly using Equation (14) as follows:

(14) $$AverageDistance=\frac{D-\beta_{1}^{d}\cdot POLLUTION}{N-\beta_{1}^{n}\cdot POLLUTION}-\frac{D}{N}$$

where N is the hour-average number of each group and D is the hour-average distance of people moving during the study period. $\beta_{1}^{n}$ is the $\beta_{1}$ when an input feature is the number of people moving in the group, and $\beta_{1}^{d}$ is $\beta_{1}$ when the feature is the corresponding distance. The function consists of two parts, where the $\frac{D-\beta_{1}^{d}\cdot POLLUTION}{N-\beta_{1}^{n}\cdot POLLUTION}$ part returns the average distance impacted by POLLUTION, and D/N part calculates the original average distance for every person in the group. The difference between these two values reflects the changing average distance that varies with pollution where N, D, $\beta_{1}^{n}$ and $\beta_{1}^{d}$ are all constants.

## 5. Results and Discussion

### 5.1. Spatial-Temporal Dataset Description

For the CDR-based people density distribution spatial scale (Figure A5), in the urban area such as Dongcheng, Xicheng Districts, the people density is much higher than the suburban area such as Huairou, Yanqing Districts, which suggests that the density decreases from the city centre to the surrounding areas. At a temporal scale, peoples’ daily activities are reduced early in the morning (e.g., 6:00 a.m., Figure A5a,e), while the density gets higher in some same urban, central, areas in the afternoon time (e.g., 6:00 p.m., Figure A5b,f).

**Figure A6:**
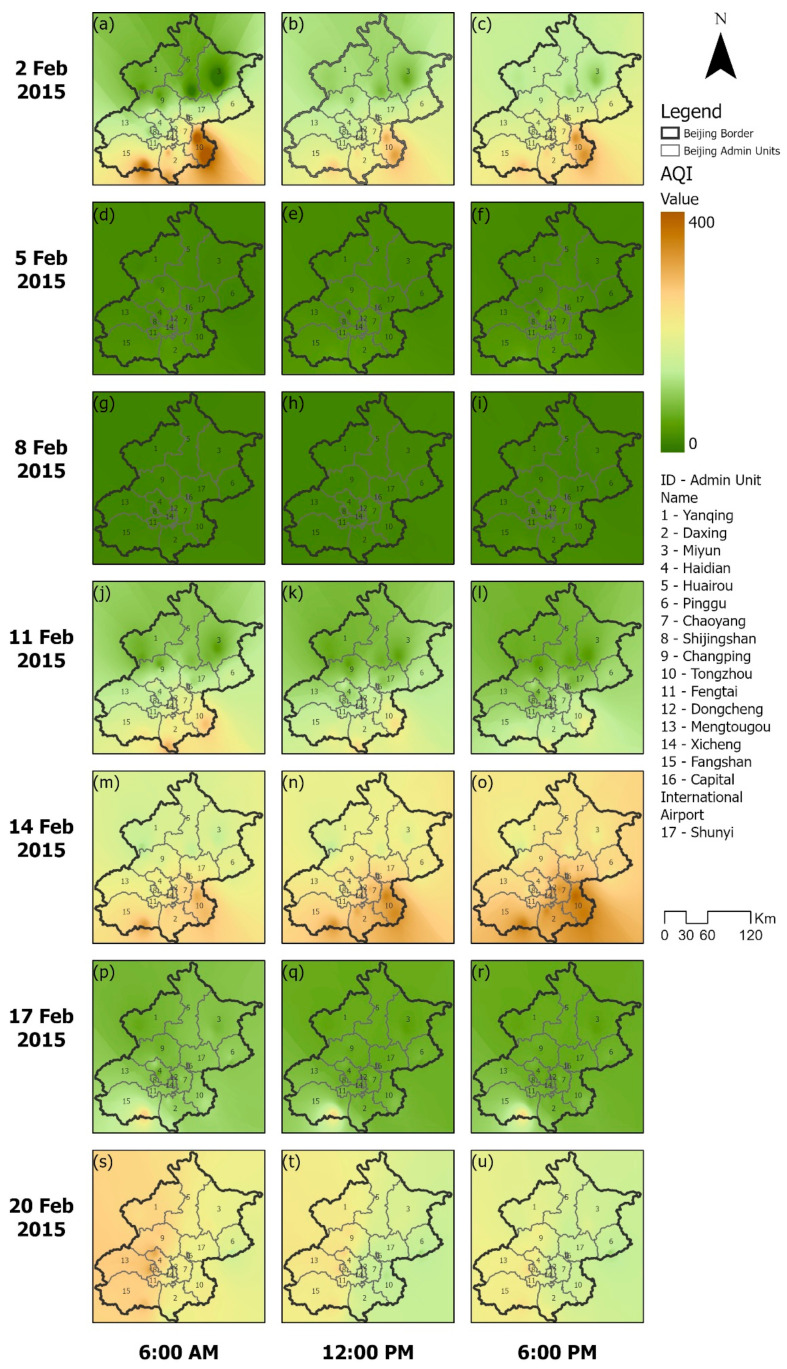
Twenty-one examples of the AQI distribution at different time points. In the layout of (**a**–**u**), every row shows the distributions of three time points: 6:00 a.m., 12:00 p.m., and 6:00 p.m. in the same day, while there are 7 days’ examples of date 2, 8, 11, 14, 17, and 20 in February 2015.

In Figure A6, it is obvious that the distributions of AQI in the study period has some irregular features. The overall trend of the AQI is from a high-value to low-value, to middle-value, to high-value, return to low-value, (Figure A6a–u), corresponding to the line chart in Figure 1. Daily, the AQI changes slightly during the morning, noon, and afternoon. However, in a few daily cases, as shown in Figure A6a–c, a slight change in air pollution (AQI > 300) from southeast to outside of Beijing is recognized. Similar patterns also happen on the 11 February (Figure A6j–l), 14 February (Figure A6m–o), 17 February (Figure A6p–r), in 2015.

**Figure A7:**
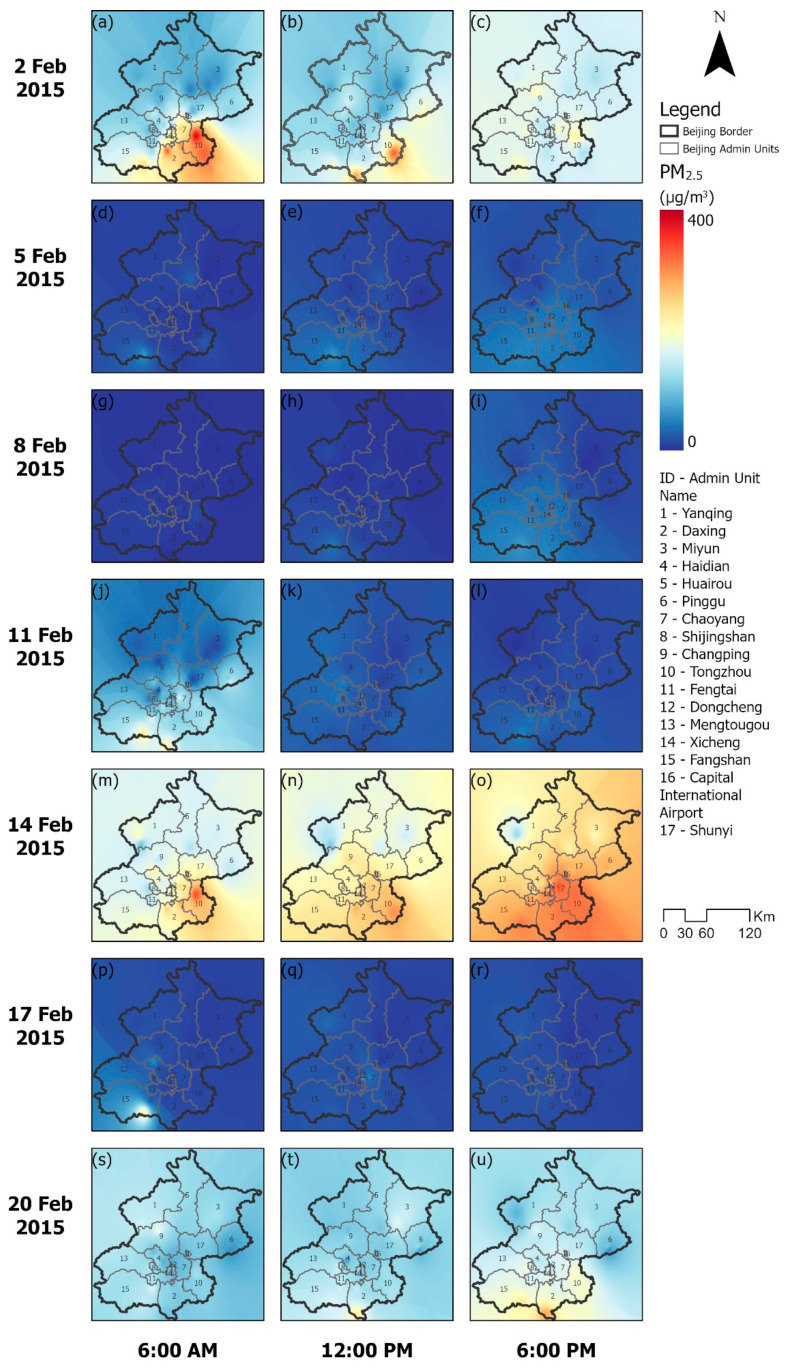
Twenty-one examples of the PM_2.5_ distribution at different time points. In the layout of (**a**–**u**), every row shows the distributions of three time points: 6:00 a.m., 12:00 p.m., and 6:00 PM in the same day, while there are 7 days’ examples of date 2, 8, 11, 14, 17, and 20 in February 2015.

The spatial-temporal distributions of PM_2.5_ are very similar to that of the AQI, especially for the overall temporal trend changes during the study period for the whole of Beijing. However, there are some daily differences between the AQI and PM_2.5_ distributions. The spatial-temporal changes in PM_2.5_ in one day is much more obvious than for the AQI. For example, on 14 February 2015, the PM_2.5_ concentration is above 300 μg/m^3^ in southeast Beijing in the morning (Figure A7m), but in the middle of the day (Figure A7n), it starts to spread to other places, resulting in the concentration in southeast Beijing decreasing to about 300 μg/m^3^ but the southwest and northeast Beijing start to suffer more serious air pollution with a concentration of PM_2.5_ above 200 μg/m^3^. Afternoons, almost all regions of Beijing have a PM_2.5_ above 200 μg/m^3^.

### 5.2. Output 1: Fixed Effect Model Results

**Figure 3:**
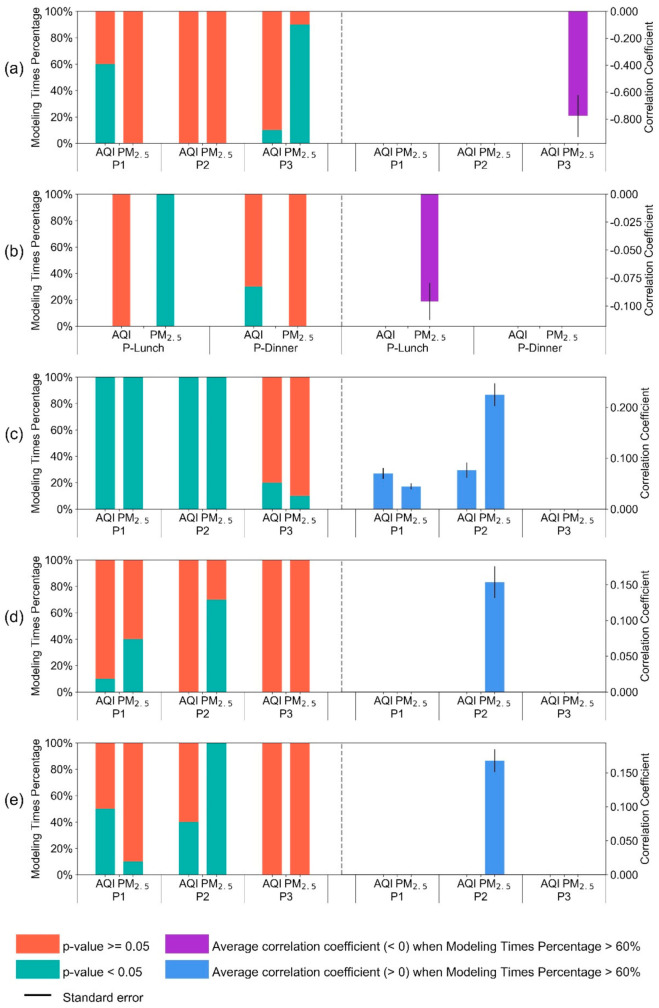
The effects of air pollution on people’s living activities. (**a**–**c**) are the results of sightseeing, eating out, and the staying-in ADLs, while (**d**,**e**) represent the use of different transportation modes ADL reflected by the situation at bus stops and subway stations. The bottom green bars in the left of each subplot show the percentage of the probability value (*p*-value) that are less than 0.05, which means the corresponding coefficients are significant within a 95% confidence interval among the 10 times they are repeated with different datasets. The red bars to the left of each subplot represent the percentage of the *p*-value is higher or equal to 0.05, which means there is no obvious relationship between the people’s activity and AQI or PM_2.5_ concentrations. We determine that PM_2.5_ as part of an AQI influences people’s activity only when the percent of significant coefficient (*p*-value < 0.05) is more than 60% (as indicated by a single length of green bar in the left graphs). We plot the mean correlation coefficient value and standard error for every group of experiments to the right of the Figures represented by blue (if the value > 0) and purple (if the value < 0) with the error bars (if it is no more than 60%, we do not plot anything in the right-side graphs). The results are reported in Table A7, Table A8, Table A9, Table A10 and Table A11.

Figure 3 documents the relationship between pollution (AQI and PM_2.5_) and people’s activities during different daily periods. According to the right part of each subplot, we see that the overall AQI, and more specifically PM_2.5_, impacts specific kinds of human activities in the three specific four-hour daytime periods. We note that in the first period (P1, 6–9 AM), air pollution has a positive influence on people staying-in (Figure 3c), which indicates people are more willing to stay in, in the morning, while the pollution conditions seem to have far less or little impact on other kinds of activities (except the dining-out activity). During the second period (P2), peoples’ activities of staying-in, using bus stops and subway stations, seem to be affected by air pollution, as shown in Figure 3c–e. For those who need to use transport, they tend to select bus and subway as their choice as they represent relatively closed-off areas that lessen the exposure to outside air pollution [43]. In P2, people tend to spend more time staying-in, at home, compared with P1. This is because period P2 covers lunchtimes, while in period P1 people generally work weekdays. In the third period (P3), air pollution impacts people who visit tourist sites, which has a negative relationship, indicating the higher the air pollution, the fewer the people who would visit these (Figure 3a). It is not hard to explain this because, since 2013, citizens living in China have improved their awareness to avoid the potential risk of illness when bad air pollution manifests itself as hazy weather (Lu et al., 2018). Air pollution tends to lower the desire of people to go to a restaurant (Figure 3b), as people may choose to cook food themselves as represented by the increasing staying-in ADL coefficient shown in Figure 3c. People eating out seem not to be impacted so much by air pollution in P3. This is after sunset when people cannot so easily visually appraise haze (in the dark). There are some differences between the overall AQI and more specifically PM_2.5_ that influence people’s activities. For example, the most significant influence is from PM_2.5_ especially in the latter part of a day (P2 and P3), while AQI’s impact is less significant and occurs mainly during P1.

### 5.3. Output 2: Spatial-Temporal Behaviour Impacting Indices of Air Pollution on ADLs

**Figure 4:**
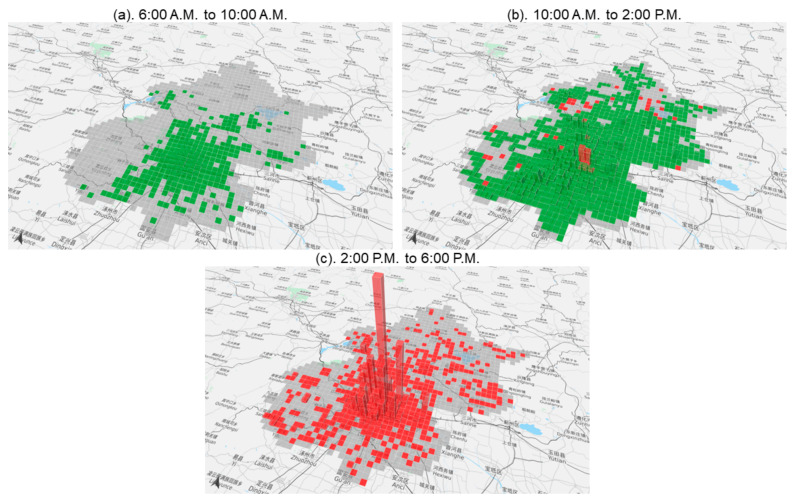
Summary behaviour impacting indices of air pollution on people’s living activity from 6:00 a.m. to 6:00 p.m. (**a**–**c**) are the spatial distributions of the influence of air pollution on all activities in period 1 (6:00 a.m. to 10:00 AM), period 2 (10:00 a.m. to 2:00 PM) and period 3 (2:00 p.m. to 6:00 PM). The green bars represent the positive effect of air pollution while the red bars represent the negative effect.

Figure 4a–c illustrate the spatial distribution of the final summary index that reflects that ADLs are affected by air pollution. Here we note that in the morning period, fewer people are impacted by air pollution for most of the days when they go to work as usual, while the green pattern means that the impact is mainly positive because staying is the main part of the index. For the middle of the day, the impacted area of air pollution starts to cover the suburbs of Beijing as shown in the greener parts in Figure 4b, w.r.t morning period. Considering that people’s activities may be affected both negatively and positively, the distribution patterns appear more complex—there could have both red and green parts at the same time. In the afternoon period, the main impacted activity is eating out, so all of the affected areas have a negative relationship with air pollution. A city centre may tend to have more accessible, well-known, frequently visited, tourist sites and entertainment sites, hence, the index is much higher than in regions away from the city centre.

The results of the map of the summary behaviour impacting indices indicate that the impact of air pollution on ADLs not only has a spatial but also a temporal, disparity. We define the no data area as an empty area disparity. These impacts appear in three different patterns temporally in one day: full positive (e.g., P1), positive and negative mixed (e.g., P2) and full negative (e.g., P3). Similarly, at the spatial scale, the impact of such patterns is also seen. For example, in the middle of Beijing, it appears to be positive in P1, then negative in P2, and still negative in P3, thus, this pattern could be classified as a positive-negative-negative (PNN) group. While in some suburbs in north Beijing (e.g., the northernmost Huairou district), the patterns include empty-positive-negative (EPN) and empty-positive-empty (EPE).

### 5.4. Output 3: Restaurant Business Loss Estimation Due to Air

The average correlation coefficient value of the random experiments’ result is −0.236 (*p* < 0.001) from the PM_2.5_ impact, which means that when PM_2.5_ increases by 1 μg/m^3^, the density of people reflected by mobile phone users’ density in the restaurant VP decreases by 0.236 people/km^2^. Thus, it is estimated that air pollution tends to cause a revenue loss for restaurants. Because we sample the mobile phone users in the first 5min of every hour and their average sample number is 1.1 million each time during daytime, we use a scaling factor to project this to the whole of the (Beijing) city population. In 2015, Beijing had 21.7 million people, so the scaling factor is roughly 20. Thus, if PM_2.5_ increases by 1 μg/m^3^, the density of actual people in the restaurant VP would decrease by 4.72 people/km^2^.

In a similar study, Zheng et al. [25] focused on how PM_2.5_ can affect people’s eating out in Beijing. They conclude that when the concentration of PM_2.5_ increased by one standard deviation, the number of people eating out decreased by 1.05%. In our case, if PM_2.5_ increases by 1 standard deviation (92.99 μg/m^3^), the density of actual people in the restaurant VP would decrease by 4.72×92.99≈438.9 person/km^2^, equal to a decrease in 10% of people eating out for lunch. The number is much higher than the study of Zheng, this may be because they combine types of eating out for breakfast, lunch and dinner, while our study only considers lunchtime and dinner. Further, another similar study, Gao et al. [24] concludes that for every 1% increase in the concentrations of PM_2.5_, the dining-out frequency of urban residents reduces 0.059% around Beijing in 2016. In our case, if PM_2.5_ increases by 1% (0.97 μg/m^3^), the density of actual people in the restaurant VP would decrease by 4.72×0.97≈4.59 person/km^2^, equal to a decrease in 0.44% for people eating out for lunch. The qualitative results of the two studies are consistent with ours.

Further, according to Equation (12), because the average area of a sampled restaurant A is 225m^2^ the CAR (using Equation (12)) could be computed as $4.72\times 20\times 225\times 10^{-6}\times 19$, roughly equal to 0.4. This means that if the PM_2.5_ increases by 1 μg/m^3^, the average revenue of one restaurant would decrease by 0.4 CNY in one day. There are several changes from a good air quality day to a polluted day in Beijing during the study period, for example, from 13 February to 14 February 2015, the AQI jumped from 108 to 248, and the PM_2.5_ concentration increases at least 125 μg/m^3^. Hence, it estimated that for example on 14 February 2015, the average revenue of one restaurant would decrease roughly by at least 50 CNY compared to February 13. In Beijing in 2015, there were 0.59 million restaurants (From 2017 China Restaurant Industry Survey Report of P.R.China. Available at http://www.chinahotel.org.cn/ChoiceOSP/upload/file/20170609/21761496997907154.pdf, accessed on 1 March 2021). When air pollution sweeps the whole city, the loss of catering could reach 29.5 million CNY for just lunchtime.

### 5.5. Output 4: Changes in the Average Distance Travelled by People Walking and Cycling

Table 6 summarises the results. More details are given in Table A17, Table A18 and Table A19. It is seen that both the numbers of people and distance of movement are impacted by AQI, negatively, when groups consist of people walking and riding (normal, manual) bikes. For the walking group, the value of the correlation coefficient between the number of them and AQI is −7.466 with a 0.031 *p*-value, while the correlation coefficient of the distance of movement and AQI is −1.201 with a 0.032 *p*-value.

Our research demonstrates that air pollution has a specific negative impact on specific transportation modes, which means that citizens already have an awareness to avoid air pollution. However, in some specific cases, people may not be able to avoid bad air pollution. Furthermore, as the number of bikes sharing schemes increases in many cities in China, this provides greater convenience for ad hoc cyclists but may also incur a financial expense. If bad air pollution arises, this may become under utilised, advertently.

Equations (15) and (16) reflect the changes in the average distance for each group w.r.t AQI changes. It is interesting to note that the relationship between these two variables is nonlinear and has a monotonically decreasing function. This means that as AQI increases, the average number of people walking and cycling decreases. For cycling, the hourly average number of people in each group is 8725 km, while the hourly-average distance moved by people is 19,276 km. The correlation coefficient result is −7.27, thus the relation between average distance for cycling group people and AQI is as follows:

(15) $$AverageDistance_{bike}=\frac{19,276-19.54\cdot AQI}{8725-7.27\cdot AQI}-\frac{19,275}{8725}$$

For the walking group, the N is 782 and D is 4485, while the correlation coefficient result is −1.201, thus the relationship between the average distance for the walking group and AQI is as follows:

(16) $$AverageDistance_{walk}=\frac{4485-7.466\cdot AQI}{782-1.201\cdot AQI}-\frac{4485}{782}$$

**Figure 5:**
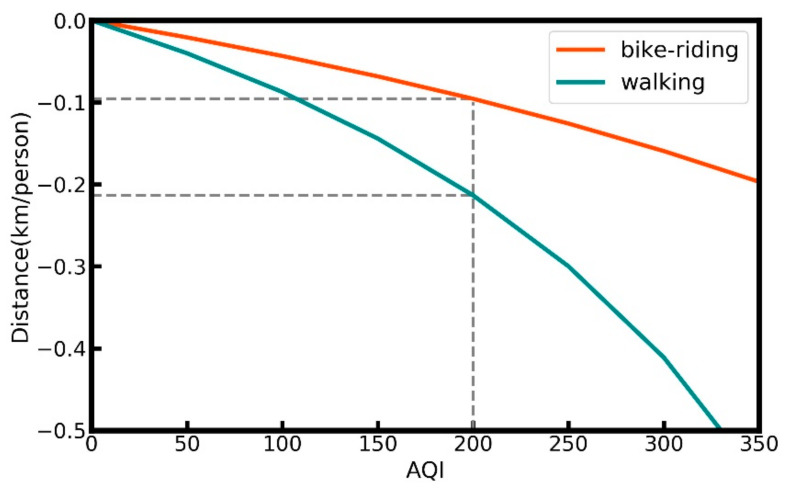
The relationships between AQI and average distance for people walking and cycling.

The curves of the two equations are shown in Figure 5 and can be used to compute how the average size of the distance moved by people cycling or walking decreases when air pollution worsens. For example, when AQI increases by 200, the cycling distance decreases by about 0.096 km, while the average walking distance decreases by 0.213 km.

Hu et al. [21] concluded that when the AQI decreases from excellent to severely polluted, the average distance of people cycling decreases by about 0.26 Km per person, while for people walking, this decreases by about 0.8 Km per person. In our case, when the AQI changes from excellent to severely polluted (AQI increases 300), the cycling distance decreases by about 0.14 Km, while the average walking distance decreases by 0.32 Km. Although the study of Hu et al. was also in 2015, the data of the study were collected from 1243 mobile application users all over China, which could explain why their results differ somewhat from ours.

## 6. Conclusions

In this study, we first define the internet of behaviours (IoB), then we apply an IoB framework to explore whether, and how, air pollution changes affect people’s specific activities of daily life quantitatively. In the IoB framework, the qualitative and quantitative impacts of air pollution on the four ADLs could give viable advice to authorities and businesses to better manage their service resources more appropriately. Our case study first provides a good application for IoB, which aims to link and analyse multiple human behaviours on mass and output this as possible feedback to the users themselves. Second, we also create a methodology that can contribute to the further development of IoB systems, frameworks, or other related components such as algorithms, communication protocols, and more diverse types of human physical behaviour detecting sensors such as millimetre wave radar, ultra wide band (UWB) and lidar.

The methodology of an IoB system presented in our study could be applied to other cities theoretically under specific conditions. These conditions are mainly related to the dataset, which is summarized as follows: Because this study focuses on people’s activities of daily living (ADL), a dataset that can estimate people’s density distribution needs to be acquired from service providers such as Telcom companies or Internet-wide service providers such as social media companies. To be more specific, a city fully covered with telecom base towers could generate the CDR data, which could be applied to compute the ADLs on mass in this study. Such data needs to be shared by a service provider but often this is regarded as a commercial product by them even for special cases such as scientific use, which is costly; sometimes only more historical rather than current data is shared. If such CDR data cannot be accessed, other geographic data with similar features (spatial and temporal resolutions, etc.) could also be used, e.g., Tencent position data (https://heat.qq.com/, accessed on 1 March 2021), Baidu heatmap (https://mtj.baidu.com/, accessed on 1 March 2021), etc. Besides the CDR data, other datasets including air pollution and weather condition datasets also need to be obtained and fused which is complex to do because data in different data sets may have different data structures, metadata, linked data and semantics. In addition, these datasets should have two dimensions (individual and time) with high spatial and temporal resolutions, to be able to be used as panel data, to apply FEMs.

An IoB framework can serve different groups of people based on their roles in society, such as citizens, governments and businesses. Hence, we propose some practical recommendations here: first, when facing the threat of bad air pollution, citizens should improve their awareness to avoid this potential great harm and take some protective measures. At the same time, as citizens, we can each increase our awareness to protect the environment, or we may face more and more environment-related threats in the future.

In terms of city authorities, besides controlling air pollution from sources such as industrial emissions, these could elect to take appropriate mitigation measures, i.e., planting more leaf or broad-leaf tree species which have been proven to have a high dust-retention capability in regions where particulate matter threatens people welfare more according to behaviour impacting indices. For example, in suburban areas with a limited green space, especially close to the bus stops or subway stations, planting high percentages of *Pinus tabulaeformis* and *Platycladus orientalis* type trees can help to clean the air.

Further, transport companies could arrange different fees for travelling at different times, such as, in peak hours, public transport ticket prices could be decreased to encourage more citizens to take public transit. Businesses could use air pollution forecasts and IoB models to conduct expedited business operations to reduce losses or gain greater profits. For example, restaurant managers could consider business solutions, such as proposing special offers at lunchtime to attract people, through calculating the costs and benefits because air pollution would decrease the number of people who want to go out for lunch. But at the same time, restaurant managers should fulfil their social responsibility of protecting citizens’ health by reminding potential customers to implement necessary measures, such as wearing a mask on the way to the restaurant. Further, because an increasing (worsening) AQI would decrease the number of people who want to cycle, as well as the average distance they ride, bike-sharing companies could adjust the charging strategy appropriately, such as reducing the cost per hour, to attract more users to ride, to reduce their potential loss in income. But in terms of their social responsibility, they could also increase the cost of riding per hour, to encourage citizens to use more public transport, to reduce their duration and exposure to air pollution outdoors.

Despite our achievements, our work still has some limitations: First, the study period and case region could be extended to detect spatial-temporal disparities. However, it is very difficult to gain access to CDR data from service providers for longer periods. The use of this methodology in other applications/studies needs a high amount of data, that maybe heterogeneous in character and may lack accessibility. Second, transport modes did not consider private cars or taxis because classifying these is difficult based upon our dataset. Third, deep machine learning could be performed to compare with the statistical models in our study to check the robustness of our study. Fourth, no quantitative comparison can as yet be performed with the work of others as, to the best of our knowledge, no one else has studied the effect of air pollution changes on a wider range of ADLs such as sightseeing, staying-in and travelling by bus or subway at this time. In the future, the methodology of the IoB system could be applied in other cities to test its robustness and to advance some of the limitations above.

## Figures and Tables

**Table 1:** Average time of main activities of residents of China in 2018.

ADL Category	Time (Minutes)	Percentage	If Is LD-ADL
**Total**	**1440**	**.**	**-**
**1. Personal Physiologically Necessary Activities**	**713**	**49.51%**	**-**
Sleeping	559	38.82%	YES
Personal Hygiene Care	50	3.47%	NO
Meals or Other Diet	104	7.22%	YES
**2. Paid Labour**	**264**	**18.33%**	**-**
Employment Work	177	12.29%	NO
Family Production and Business Activities	87	6.04%	NO
**3. Unpaid Work**	**162**	**11.25%**	**-**
Housework	86	5.97%	YES
Accompanying and Caring for Family	53	3.68%	NO
Purchase Goods or Services (including Medical Treatment)	21	1.46%	NO
Charitable Activities	3	0.21%	NO
**4. Personal Discretionary Activity**	**236**	**16.39%**	**-**
Fitness Exercise	31	2.15%	YES
Listening to Radio or Music	6	0.42%	NO
Watching TV	100	6.94%	YES
Reading Books, Newspapers and Periodicals	9	0.63%	NO
Leisure and Entertainment	65	4.51%	NO
Social Interaction	24	1.67%	NO
**5. Learning and Training**	**27**	**1.88%**	NO
**6. Transportation**	**38**	**2.64%**	YES
**Other: Use the Internet**	**162**	**11.25%**	NO

**Table 2:** Abbreviations and explanations.

Abbreviation	Explanation	Abbreviation	Explanation
ADL	Activities of daily living	MEP	Ministry of Environmental Protection
AQ	Air quality	MPUD	Mobile phone users’ density
AQI	Air quality index	NCDC	National Climatic Data Centre
CAR	Change in average revenue	NOAA	National Oceanic and Atmospheric Administration
CDR	Call Detail Record	OLS	Ordinary Least Squares
CI	Cell Identity	P1	Period 1
CNAAQS	Chinese National Ambient Air Quality Standard	P2	Period 2
CNY	Chinese Yuan	P3	Period 3
CSV	Comma-Separated Values	PCC	Per Capita Consumption
CTRM	Comparison Test of Reference Method	P-Dinner	Period dinner
EDR	Effective Data Rate	P-Lunch	Period lunch
EPE	Empty-positive-empty	PM	Particulate Matter
EPN	Empty-positive-negative	PNN	positive-negative-negative
FEM	Fixed Effect Model	POI	Point of Interest
GIS	Geographic Information Science	PoM	Parallelism of Monitors
GSM	Global System for Mobile Communication	PURT	Panel Unit Root Test
HT	Harris-Tzavalis	S1	Strategy 1
IDW	Inverse Distance Weighting	S2	Strategy 2
IMSI	International Mobile Subscriber Identification Number	SIM	Subscriber Identity Module
IoB	Internet of Behaviours	SPSA	Specific place with a specific activity
IoT	Internet of Things	TEOM	Tapered Element Oscillating Microbalance
IPS	Im-Pesaran-Shin	UWB	Ultra Wide Band
KDE	Kernel Density Estimation	VP	Voronoi polygon
LD-ADL	Location-driven ADL		

**Table 3:** A summary of related works.

Author(s)	Detected ADL(s)	Data Collection	Analysis Method	Limitation(s)
De Freitas [16]	Beach user behaviour	Questionnaire survey	Two-dimensional regression analysis	Single ADL; Traditional survey method
Lin et al. [17]	Stay in behaviours of elder people	Multi-sensors	Traditional machine learning methods	Single ADL; Small spatial scale;
Jiang et al. [18]	Maximum number of park visits	On-line and off-line survey	Quantile regression analysis	Single ADL; Small spatial scale; cannot consider unobservable variables
R-Toubes et al. [19]	Tourist number on beaches	Webcam images in combination with real-time weather	Pearson relatsionship anaylsis	Single ADL; Small spatial scale; cannot consider unobservable variables
Zhao et al. [20]	Cycling behaviour	Survey in various locations during different periods	Conceptualize the relationship via perceptions	Single ADL; Small spatial scale; cannot consider unobservable variables
Hu et al. [21]	Outdoor exercise (running, biking, and walking)	APP Tulipsport users’ data	Multivariate analyses of variance	Too few samples; cannot consider unobservable variables
Gao et al. [24]; Zheng et al. [25]	Dining-out activities	Third-party website (dianping.com), (accessed on 26 July 2021)	FEMs	Data objectivity and model robustness have not been tested

**Table 4:** Parameters and specifications of the sensors to collect data.

Dataset	Sensor	Range	Resolution	Accuracy
PM_2.5_	PM_2.5_	0~10,000 μg/m^3^	0.1 μg/m^3^	≥85%
PM_10_	PM_10_	0~10,000 μg/m^3^	0.1 μg/m^3^	≥85%
SO_2_	SO_2_	0~500 ppb	0.1 μg/m^3^	±2%F.S.
O_3_	O_3_	0~500 ppb	0.1 μg/m^3^	±4%F.S.
NO_2_	NO_2_	0~500 ppb	0.1 μg/m^3^	±2%F.S.
CO	CO	0~50 ppm	0.1 mg/m^3^	±2%F.S.
Temperature	Thermometer	−50~50 °C	0.1 °C	±0.2 °C
Wind	Wind Speed	0~60 m/s	0.1 m/s	±(0.5 m/s + 0.03 v)
Cloud Amount	-	0~100%	-	-
Precipitation (Rain)	Tipping-Bucket Rain Gauge, Weighing Precipitation	≤4 mm/min	0.1mm	±0.4 mm (≤10 mm), ±4% (>10 mm)
Snow (depth)	Automatic Snow Depth Observation Instrument, Ultrasonic or Laser Sensors	0~2000 mm	1 mm	±10 mm

**Table 5:** Variable definitions and summary statistics.

Variable	Definition	Obs.	Mean	Std.
Mobile phone users’ density (MPUD) variables (Person/Km^2^)				
Sightseeing	MPUD for the sampled sightseeing POIs	92,232	529.287	469.586
Eating out	MPUD for the sampled restaurant POIs	95,256	519.974	440.782
Stay in	MPUD for the sampled house POIs	100,593	539.684	452.293
Bus Stop	MPUD for the sampled bus stop POIs	49,811	562.951	418.586
Subway Station	MPUD for the sampled underground station POIs	49,310	620.532	509.309
Sources: CDR data is from China Mobile Limited Company (Beijing Branch), POI dataset is from AutoNavi Software Limited Company.				
Pollution variables				
AQI	Hourly air quality index	17,640	153.025	93.099
PM_2.5_	Hourly PM_2.5_ concentration (μg/m^3^)	17,640	107.929	89.774
Source: Ministry of Environmental Protection of the People’s Republic of China				
Weather variables				
TEMP	Mean temperature of the site in Beijing (°F)	504	34.381	8.642
WIND	Mean wind speed of the site in Beijing (m/s)	504	6.871	5.412
CLOUD	Cloud coverage score (0 to 3), 3 = full, 0 = none	504	0.325	0.741
RAIN	One-hour liquid precipitation of the site (inches)	504	0	0
SNOW	One-hour snow depth of the site (inches)	504	0	0
Source: Daily weather data are collected from the National Oceanic and Atmospheric Administration				
Type of day variables				
Spring Festival	1 = today is in Spring Festival Holiday, 0 = otherwise	-	-	-
Weekend	1 = today is weekend, 0 = otherwise	-	-	-
Valentine’s Day	1 = today is Valentine’s Day, 0 = otherwise	-	-	-
Source: None				

Note: Obs. refers to the number of observations. Std. refers to standard deviation.

**Table 6:** The effects of air quality on walking and cycling people.

	Number of People		Distance of Movement	
	AQI	PM_2.5_	AQI	PM_2.5_
Walk	−7.466 *	−0.661	−1.201 *	−0.207
	(0.031)	(0.782)	(0.032)	(0.608)
Riding bike	−19.540 *	−2.839	−7.271 *	−1.015
	(0.028)	(0.665)	(0.044)	(0.715)

Note: The dependent variable is the number of people who ride electronic bikes or their moving distance hourly in Beijing. *p*-values are reported in parentheses; * = *p* < 0.05.

**Figure A1:**
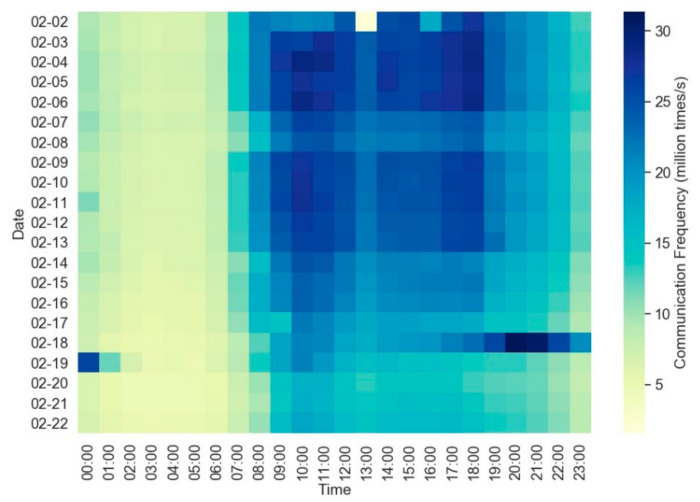
Hourly distribution of CDR number over the whole study period.

**Figure A2:**
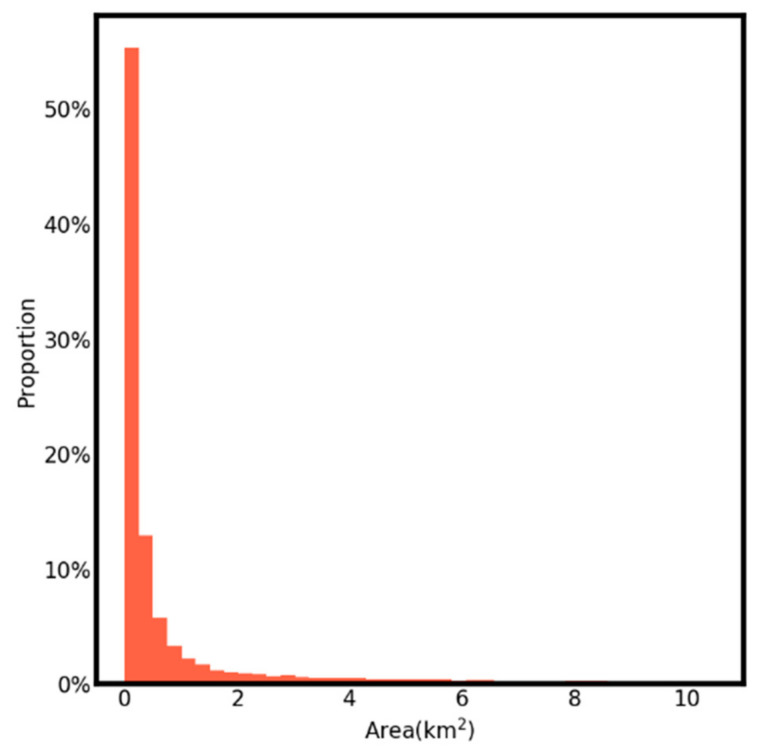
Areas proportion distribution of base station VP.

**Figure A3:**
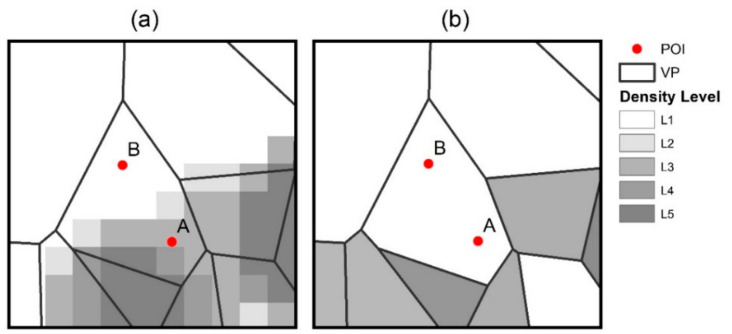
Comparison of two strategies of using density distributions. (**a**) illustrates an example of S2, while (**b**) shows the corresponding case of S1.

**Figure A4:**
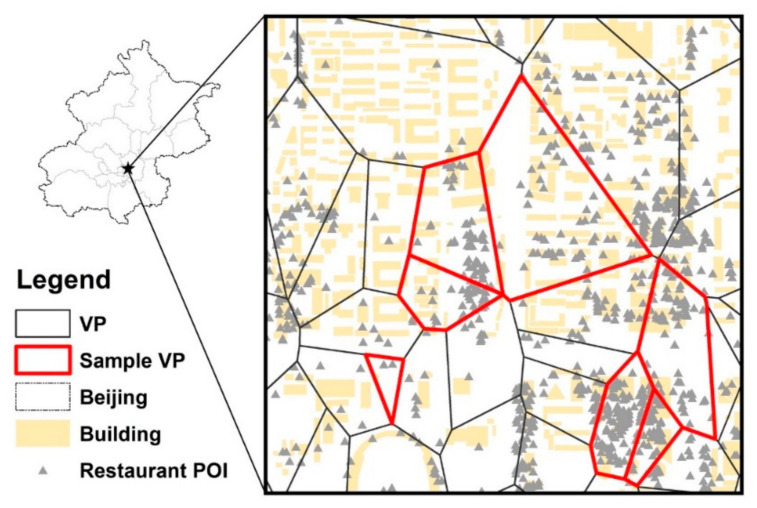
Restaurants Community (RC) example for the Sanlitun area of Beijing.

## Data Availability

Publicly available datasets were analysed in this study. These datasets can be found here: Air pollution data: http://www.cnemc.cn/, (accessed on 1 March 2021); Weather data: https://www.ncdc.noaa.gov/, (accessed on 1 March 2021). The CDR, POI, and Building distribution datasets are available on request from the corresponding author. The data are not publicly available due to privacy.

## Appendix A. Additional Description of Datasets

### Appendix A.1. Additional Materials for CDR Dataset Description

A CDR contains: The International Mobile Subscriber Identification Number (IMSI) that is a unique code for every subscriber identity module (SIM) card to identify users on the network; a timestamp that records when interactive communication events happen; Cell Identity (CI) corresponding to the base station location (Table A1). CDRs are generated every 1 s (its temporal resolution of recording) and stored in a Comma-Separated Values (CSV) file.

**Table A1:** Recorded data structure.

ID	Name	Description
**1**	Timestamp	Interactive time of users and base station
**2**	CI	Corresponding base station’ identity
**3**	IMSI	The encrypted ID of users

We used the locations of all the 51,216 mobile base stations shown in the Global System for Mobile Communication (GSM) engineering parameters inner structure (Table A2). However, because some base stations are so close that the identified latitude and longitude are practically the same, we combined “collocated” base stations such that their number reduces by about ⅔ from 51,216 to 17,445. The coverage area of each mobile base station can be approximated as a Voronoi polygon (VP) that is built around it (Thiessen, 1911). When a phone is used to make a call or send a text message, its location is found via being in range of the specific mobile base station.

**Table A2:** The inner structure of GSM engineering parameters.

ID	Name	Description
**1**	CI	Unique ID of the base station
**2**	Lat, Lon	Latitude and longitude of the base station location

Figure A1 illustrates more details for hourly record number information, from which we observe that for most days, numbers of records increase suddenly from 6 a.m. These also become minimal from 11 p.m., which is because people call or text much less at night when most people are asleep. However, during the daytime, the number of records reflect better the active level of people’s activity.

### Appendix A.2. Error Analysis When Estimating Mobile Phone Users’ Density Distributions

In many cities such as Beijing, the use of mobile phones in an urban area is larger than in a suburban one and the density of the base stations is larger, while in a suburban area, a single station covers a bigger circle area impacted by the requirement and terrain. Figure A2 shows the distribution areas of the base stations. It can be observed that more than 50% of areas of base stations are less than 0.25 Km^2^, corresponding to a 500 m positioning resolution if we represent or abstract the base station as a square. In Beijing, almost 80% of people live in the urban area, which is covered by a dense mass of base stations so that we can regard CDR-based positioning accuracy as 500 m.

Then we are concerned if the resolution of the CDR-based positioning density distribution is accurate enough to let the extracted value of a vector point accurately represent people’s density or other feature values for the POI.

A POI is a random sample point in a region because a vector point cannot represent an area, for example, a restaurant POI does not mean this restaurant has only one sampled point. In terms of the sightseeing ADL, these areas consist of sites such as parks that are used for touring and for leisure and are larger than 1 Km^2^. Community regions have a similarly large average area as well. In terms of the transport mode options, except for people walking and bike-riding, we focus on people who intend to take a bus and the subway. Bus stops and subway stations are the POIs that we use to represent the people’s density values to investigate any potential changes in these concerning air pollution. According to the statistics from a route planning website (https://lbs.amap.com/getting-started/path, accessed on 1 March 2021), the average distance between bus stops in Beijing is over 1 Km, which is twice as long as the KDE density distribution with a 500 m spatial resolution in the main urban area. Note, the average distance between two adjacent subway stations in Beijing is about 1.5 Km [44], which is much larger than 500 m. Furthermore, the transport bus and subway POIs sites tend to be set beside main roads in Beijing. There are fewer other types of POI close by, such as restaurants or apartments, so we can use these POIs to collect information on how people move by bus and subway. Thus, these POIs can represent the situation for these two kinds of activity. However, in terms of the eating out option, some restaurants may be part of a big mall, mixed in amongst other kinds of shops such as clothing stores representing other human activities. Using only a single POI (only eating out) to represent here may cause a bigger error. To solve this problem, we present an additional strategy to decrease the error and test for this in the model and define it as Strategy 2 (S2) while the previous one is defined as S1. The details of S2 are introduced in Appendix B.

### Appendix A.3. Error Control Solution When Estimating Mobile Phone Users’ Density Distributions

To decrease this unknown impact, we present a standard method for spatial resolution as follows. First, we randomly generate an equal number of points in each VP that is equal to the actual records of sampled mobile phone users in every corresponding VP, where every point represents one mobile phone user. Then, we use the kernel density estimation (KDE) method to estimate hourly density distributions for the whole of Beijing, with the raster resolution parameter set as 500 m. The final distribution raster maps the datasets for continuous hours with a geospatial resolution of 500 m × 500 m. Although the errors could still be spatially uneven, this method can reduce the error when extracting values (i.e., mobile user density, PM_2.5_ concentration, etc.). POI, especially when the POI is within a bigger VP. For example, Figure A3a shows the density distribution using KDE, while Figure A3b shows a simple symbolization method to display the density for every VP. we divide the density into five levels in this case and as the level index increases, the density increases. POI A and B are located at the same VP on different sides of it. In (b) the original density distribution, A and B have the same density value; however, because of the spatial autocorrelation theorem, A should have the value closer to level 4 or level 5. Hence, after the KDE process stage, point A get a value at density level 3, which is more accurate.

### Appendix A.4. Description of Other Datasets

#### Appendix A.4.1. Additional Materials for Air Quality Datasets

Throughout this paper, we study the role of air pollution on people’s activities. We recognize that there are several pollutant criteria. We have emphasized the central role of PM_2.5_ both because we observe this variable’s value by POI/hour and because several independent research studies have documented its role in raising the mortality and morbidity risk, e.g., [45,46]. Our focus is on the Air Quality Index (AQI) and concentrations of key air pollutants (PM_2.5_, PM_10_, SO_2_, O_3_, NO_2_, CO) and their correlations for Beijing, see Table A3.

Scientific studies show that high concentrations of a particular matter can cause severe air pollution problems in some Chinese cities in recent years [47,48]. The Clean Air Alliance of China Clean Air Management Report in 2016 (CAAC Clean Air Management Report. Bulletin on Clean Air Alliance of China in 2016. Available at http://www.cleanairchina.org/file/loadFile/145.html, accessed on 1 March 2021) stated that particulate matter was still the main factor of air pollution in China in 2015 and that Beijing was (and still is) one of the most polluted cities for PM_2.5_ and PM_10_ in China. Besides these two pollutants, O_3_ and NO_2_ emissions still exceed the standard from the Chinese National Ambient Air Quality Standard (CNAAQS) (Chinese National Ambient Air Quality Standard. Bulletin on the Ministry of Ecology and Environment of the P.R. China in 2012. Available at http://kjs.mee.gov.cn/hjbhbz/bzwb/dqhjbh/dqhjzlbz/201203/W020120410330232398521.pdf, accessed on 1 March 2021), which is also reflected in the study period for 1.8% of the hours for O_3_ and 15.5% for NO_2_. According to MEP’s AQI data, PM_2.5_ was the primary pollutant for 64.3% of the hours and PM_10_ was in 18.7% of hours the major polluting factor in our study period.

People can visually perceive visible particulate matter in the air, thus they perceive PM_2.5_ and PM_10_ with their eyes. SO_2_ is an odorous gas that is emitted with industrial smoke and other coloured sulphides; people can see and smell it at high concentrations. However, during this study period, its concentration was low enough not to be perceivable. As ground-level O_3_ and CO are both invisible and odourless; people tend to be less likely to perceive their effects. NO_2_ was always at a low concentration level during the study. However, it reacts with some organic compounds in the air to increase other pollutants such as O_3_ and PM_2.5_, which means it may be more indirectly, rather than be directly perceived (see Table A3). Also note the individual elements of AQI seem to be highly correlated with PM_2.5_, except for SO_2_ and CO, which are consistently low. PM_2.5_ is highly correlated with PM_10_ (correlation coefficient = 0.703, *p* < 0.001), AQI (correlation coefficient = 0.625, *p* < 0.001) and NO_2_ (correlation coefficient = 0.723, *p* < 0.001). In contrast, O_3_ is negatively correlated with PM_2.5_. Thus, PM_2.5_ is the primary pollutant for the majority of days in Beijing during our study period.

**Table A3:** Air pollution Statistics Based on Beijing data.

	AQI	PM_2.5_	PM_10_	SO_2_	O_3_	NO_2_	CO
Mean concentration (μg/m^3^)	/	107.929	133.959	32.307	39.289	53.460	1.698
% hours when it is the primary pollutant	/	64.29%	18.65%	0.00%	1.79%	15.48%	0.00%
Whether it is easily perceived	/	YES	YES	YES	NO	NO	NO
Correlation between it and PM_2.5_	0.625 ***	/	0.703 ***	0.760 ***	-0.626 ***	0.723 ***	0.832 ***

Note: *** *p* < 0.001.

In terms of particulate matter (PM), PM_10_ and PM_2.5_ are collected by a continuous monitoring system that consists of a sample acquisition unit, sample measurement unit, data acquisition and transmission unit and other auxiliary equipment. The measuring methods of the monitoring instruments configured in the system are the β-Ray absorption method and tapered element oscillating microbalance (TEOM) method, which are performed in a PM_2.5_ sampler or PM_10_ sampler. The principles and operation details of the two methods are specified in the related standards, which can be accessed from the National public service platform for standards information (China) (http://std.samr.gov.cn/, accessed on 1 March 2021) (Note all the specifications or standards in this paper refer to this platform).

Range and resolutions are also key parameters when collecting data using sensors, which are described in Table A4. Furthermore, the accuracy and repeatability of the data collection, reflected by the use of parallelism of monitors (PoM), effective data rate (EDR) and comparison test of reference method (CTRM) are reported in Table A4. The definitions of the indicator are as follows.

PoM: Root mean square of each batch data result: In the same test environment, adjust the inlet of the three monitors to the same height, and the distance between the monitors is 2–4 m. After the calibration and setting of the sampling flow, the instrument parallelism test is carried out.

EDR: After debugging, the monitor will run continuously for at least 90 days to test the effective data rate. During this period, the maintenance time and details are recorded, and the daily average value of the three monitors to be tested, are analysed.

CTRM: At least three samplers are used for the reference method, meanwhile, an automatic testing monitor works simultaneously. The automatic monitoring data C and the reference method test data r in the same sampling period are taken as a data pair, and a total of 10 groups of samples are tested. Then the reference test data and the corresponding automatic monitoring data are analysed using linear regression, and the slope k, intercept B and correlation coefficient r of the test regression curve are analysed.

Here we list all the referred specifications or standards in Table A4:

- HJ 655-2013 (China): Technical Specifications for Installation and Acceptance of Ambient Air Quality Continuous Automated Monitoring System for PM_10_ and PM_2.5_
- HJ 653-2013 (China): Specifications and Test Procedures for Ambient Air Quality Continuous Automated Monitoring System for PM_10_ and PM_2.5_
- HJ 93-2013 (China): Specifications and Test Procedures for PM_10_ and PM_2.5_ Sampler

**Table A4:** Parameters and Specifications of the sensors to measure PM_10_ and PM_2.5_.

PM	Sensor	Range	Resolution	CTRM	EDR	PoM	Specification
PM_2.5_	PM_2.5_ Sampler	0~10,000 μg/m^3^	0.1 μg/m^3^	*Coef.* ≥ 0.93	≥85%	≤15%	(1) HJ 655-2013 (2) HJ 653-2013 (3) HJ 93-2013
PM_10_	PM_10_ Sampler	0~10,000 μg/m^3^	0.1 μg/m^3^	*Coef.* ≥ 0.95	≥85%	≤10%	

Note: Coef. indicates the coefficient in the linear regression results of a CTRM.

In terms of the other four pollutants, the monitoring system consists of the sampling device, calibration equipment, analytical instrument, data acquisition and transmission equipment. The system collects the pollutants data using a point analyzer, which refers to the monitoring and analysis instrument that collects the ambient air through sampling the concentration of an air pollutant at a fixed point.

The measurement parameters, such as the measurement range and the sensor resolution, and the sensors themselves used to measure each pollutant are shown in Table A5. The indication error represents the accuracy of the collected data, which is defined as follows After the monitoring system runs stably, a zero-point calibration and full-scale calibration are carried out respectively, a standard gas with a concentration of about 50% of the range is introduced, and the display value is recorded after the reading is stable; Then a zero calibration gas is injected. The test is repeated three times, and the indication error of the analytical instruments are calculated according to the formulae given in specifications.

The reference standards used are given below:

- HJ 193-2013 (China): Technical Specifications for Installation and Acceptance of Ambient air Quality Continuous Automated Monitoring System for SO_2_, NO_2_, O_3_ and CO
- HJ 654-2013 (China): Specifications and Test Procedures for Ambient Air Quality Continuous Automated Monitoring System for SO_2_, NO_2_, O_3_ and CO

**Table A5:** Parameters and Specifications of the sensors to measure SO_2_, NO_2_, O_3_ and CO.

Pollution	Sensor	Measure Method	Range	Resolution	Indication Error	Specification
SO_2_	SO_2_ Analyzer	Ultraviolet Fluorescent method	0~500 ppb	0.1 μg/m^3^	±2%F.S.	(1) HJ 193-2013 (2) HJ 654-2013
O_3_	O_3_ Analyzer	Ultraviolet Absorbance method	0~500 ppb	0.1 μg/m^3^	±4%F.S.	
NO_2_	NO_2_ Analyzer	Chemiluminescence Detection method	0~500 ppb	0.1 μg/m^3^	±2%F.S.	
CO	CO Analyzer	Non-dispersive Infrared Absorption method, Gas Filter Correlation Infrared Absorption method	0~50 ppm	0.1 mg/m^3^	±2%F.S.	

Note: ppb: parts per billion; ppm: parts per million; F.S. indicates full scale.

#### Appendix A.4.2. Weather Conditions

Weather data are collected from the National Oceanic and Atmospheric Administration (NOAA). The data are collected from weather stations included in the National Climatic Data Centre (NCDC) of NOAA (https://www.ncdc.noaa.gov/, accessed on 1 March 2021). The temporal resolution of the weather data is hourly, but in space, they are from only one pollution monitoring station because the weather conditions do not vary across Beijing at the same time. Most of our POIs distribution is in the core area of Beijing, where the temperate and other weather conditions vary less across this area, which has little influence on the regression results. It is worth mentioning that in our 21-day study period, the rain and snow value are all zero. Hence, in this study, we considered the weather factors to only include temperature, wind speed and sky/cloud cover.

Weather sense is governed by international standards. The key parameters of the meteorological sensors to collect corresponding data are shown in Table A6, including the range and resolution, accuracy. Measurements made using scientific instruments are repeatable as evidenced by the observation that readings don’t change when weather patterns are stable.

Standard specifications can be downloaded from national public bodies according to a unique identifier as follows:

- GB/T 35221-2017 (China): Specification for surface meteorological observation—General
- GB/T 35226-2017 (China): Specification for surface meteorological observation—Air temperature and humidity
- GB/T 35227-2017 (China): Specification for surface meteorological observation—Wind direction and wind speed
- GB/T 35222-2017 (China): Specification for surface meteorological observation—Cloud
- GB/T 35228-2017 (China): Specification for surface meteorological observation—Precipitation
- GB/T 35229-2017 (China): Specification for surface meteorological observation—Snow depth and snow pressure

**Table A6:** Parameters and Specifications of the sensors to measure weather conditions.

Weather Condition	Sensor	Range	Resolution	Accuracy	Specification
Temperature	Thermometer	−50~50 °C	0.1 °C	±0.2 °C	GB/T 35226-2017
Wind	Wind Speed Sensor	0~60 m/s	0.1 m/s	±(0.5 m/s + 0.03 v)	GB/T 35227-2017
Cloud Amount	-	0~100%	-	-	GB/T 35222-2017
Precipitation (Rain)	Tipping-Bucket Rain Gauge, Weighing Precipitation Sensor	≤4 mm/min	0.1 mm	±0.4 mm (≤10 mm), ±4% (>10 mm)	GB/T 35228-2017
Snow (depth)	Automatic Snow Depth Observation Instrument, Ultrasonic or Laser Sensors	0~2000 mm	1 mm	±10 mm	GB/T 35229-2017

Note: The accuracy is defined as the maximum allowable error of the specifications.

Note that in terms of determining the Cloud coverage in the sky, this is often determined from visual measurements and image analysis and may even be determined manually.

(1) POI

To study how SPSAs might be impacted by air pollution, we analysed this relationship for four situations: if people go out sightseeing, if people eat out, their transport mode options and if they stay in options. The point of interest (POI) for every situation are different datasets that were obtained from the AutoNavi Software Limited Company (https://mobile.amap.com/, accessed on 1 March 2021). In terms of sightseeing, we consider whether or not the place is free (of charge) to visit as this could influence whether people go to visit them or not. We extract 200 sightseeing POIs that are free, such as Chaoyang Park, Nanluoguxiang and Tiananmen Square, and 200 POIs where citizens need to pay to visit, such as Lama Temple, The Summer Place and Yuyuantan Park. For the eating out option, 200 restaurant POIs in Beijing have been identified or extracted. We also extract 200 house or community POIs to study the impact of air pollution. To reflect people’s transport mode options, we selected bus stops and subway stations as POIs (linked to the use of bus and subway as commonly used transport modes) that are static or fixed positions or waypoints during citizens’ use of transport. For these two kinds of transport mode POIs, we select or extracted 100 of each to a total of 200 POIs. It is important to note that sightseeing POIs can represent at least 1 Km^2^ of the area around the points, which are also called buffer zones. This decreases the error relating to the limit of CDR-based positioning accuracy, which is analysed in more detail in Appendix A.2. Note also that all POIs are extracted, spatially randomly, which means they have a very dispersed spatial distribution for each type of POI.

(2) Building distribution

Building spatial distribution data is represented as ESRI polygon data in shapefile format (https://www.esri.com/library/whitepapers/pdfs/shapefile.pdf, accessed on 1 March 2021). This data covers the main urban area defined to be within the sixth ring road in Beijing. The primary use of this dataset is to extract the area of a building within a single base station VP (S2, Appendix B), and then to calculate the proportion of a restaurant area in this part of a building (Section 4.2.4).

## Appendix B. An Additional Strategy (S2) for the Eating out ADL

Although in some areas such as large shopping malls, restaurants may be scattered amongst other kinds of shops, we can just select those specific base stations where the POIs nearby mainly consist of restaurants. We define a restaurant community (RC) area using the following rules. If a base station VP has N number restaurants while the building area in this region is B Km^2^, and they then meet the following condition N×A>B×50%, where A is the minimum area of 80 m^2^ for a restaurant based on Beijing catering enterprise operating area access standards in 2007, the base station VP is an RC. It is worth mentioning that we assume a restaurant with an 80 m^2^ area is too small to have more than one floor. Based upon this rule, and my building distributions ever and POIs dataset, we sample 71 RCs as an additional area to study. For example, Figure A4 shows the Sanlitun area, which is one of the most famous business districts in Beijing, but only the VP that meets the RC judging condition would be sampled, as shown by the red outlines. Then, we extract the average density values, air quality data and weather condition data over the polygon, and finally, we still use the same FEM to analyse the relationship between the air quality and the eating out the option of people.

## Appendix C. Additional Materials for Results

### Appendix C.1. Spatial-Temporal Distributions for Key Datasets

This appendix aims to describe the spatial-temporal characteristics of the CDR-based people density distribution, AQI and PM_2.5_ IDW result in the study period and region. Because every hour during the 21 days’ daytime has one distribution per kind of dataset, there are more than 2500 distribution maps. Thus, we only plot some examples for three key datasets, CDR-based people density, AQI and PM_2.5_, as sampled distributions and describe the characteristics for their spatial and temporal features here.

### Appendix C.2. The Results of FEM Regressions

We report the FEM results for different POIs in different periods in a day in Table A7, Table A8, Table A9, Table A10, Table A11, Table A12, Table A13, Table A14, Table A15 and Table A16 (using Equation (8) in the main manuscript). Table A7, Table A8, Table A9, Table A10 and Table A11 show the 10 times of the conducted random experiments (see Section 4.2.2 in the main text) involving sightseeing, eating out/restaurant, staying-in and travelling via bus stop and subway station, POIs. Columns (i), (iii) and (v) in Table A7, Table A9, Table 10, Table 11 show the impact of the AQI on people’s activities in three periods that have been defined in the main text. Columns (ii), (iv) and (vi) illustrate the impact of the PM_2.5_ concentration on people’s activities. Table A8 shows this for the eating out POI. It has a similar format to the other 4 POIs but just has 4 columns because it only includes the two time periods used mostly for eating. In Table A8, the correlation coefficient between AQI and mobile phone users’ density (MPUD) in the lunch period (11 AM to 2 PM) and the dinner period (5 PM to 8 PM), are reported in columns (i) and (iii), while the MPUD coefficient with PM_2.5_ is reported in columns (ii) and (iv). Table A12, Table A13, Table A14, Table A15 and Table A16 show the results of the FEMs that use all POIs, where the correlation coefficients are utilized to compute the spatial behaviour impacting indices in Section 4.2.3 of the main text. Table A12, Table A13, Table A14, Table A15 and Table A16 have the same structure as Table A7, Table A8, Table A9, Table A10 and Table A11.

**Table A7:** Ten times of the effect’s estimation of air pollution on people’s activity on the randomly sampled sightseeing POIs.

	(i—AQI)	(ii—PM_2.5_)	(iii—AQI)	(iv—PM_2.5_)	(v—AQI)	(vi—PM_2.5_)
Times	Period1	Period1	Period2	Period2	Period3	Period3
1	0.0741 *** (0.001)	0.0215 (0.117)	0.0005 (0.988)	−0.0006 (0.988)	0.0854 (0.63)	−0.6276 *** (0.001)
2	0.0289 (0.057)	0.0067 (0.554)	−0.0065 (0.848)	−0.0153 (0.693)	0.1172 (0.512)	−0.9823 *** (0)
3	0.0551 ** (0.006)	−0.0011 (0.93)	−0.0113 (0.752)	−0.0462 (0.262)	−0.0747 (0.719)	−0.8426 *** (0)
4	0.0402 * (0.039)	0.0001 (0.991)	−0.0102 (0.781)	−0.0082 (0.83)	0.1253 (0.555)	−0.5164 (0.056)
5	0.0296 (0.105)	−0.0073 (0.568)	0.0192 (0.56)	0.0084 (0.804)	0.2183 (0.284)	−0.5702 * (0.043)
6	0.0511 * (0.023)	0.0138 (0.297)	−0.0065 (0.858)	0.0314 (0.402)	0.1038 (0.623)	−0.7019 ** (0.003)
7	0.0428 * (0.017)	0.0067 (0.615)	−0.0026 (0.942)	−0.0149 (0.723)	0.3725 * (0.034)	−0.6325 ** (0.004)
8	0.0276 (0.146)	−0.0051 (0.678)	−0.0112 (0.735)	−0.0183 (0.603)	0.1539 (0.382)	−0.7492 ** (0.002)
9	0.0301 (0.076)	−0.0043 (0.732)	0.0142( 0.627)	−0.0014 (0.966)	−0.0336 (0.874)	−0.9454 *** (0.001)
10	0.0596 *** (0.001)	0.0111 (0.415)	0.0237 (0.501)	−0.0056 (0.889)	0.0243 (0.887)	−0.9433 *** (0)

Note: * *p* < 0.05, ** *p* < 0.01, *** *p* < 0.001.

**Table A8:** Ten times of the effect’s estimation of air pollution on people’s activity on the randomly sampled restaurant POIs.

	(i—AQI)	(ii—PM_2.5_)	(iii—AQI)	(iv—PM_2.5_)
Times	Period Lunch	Period Lunch	Period Dinner	Period Dinner
1	0.0023 (0.94)	−0.0946 *** (0)	0.4986 * (0.022)	−0.1509 (0.647)
2	0.0167 (0.528)	−0.0733 *** (0)	0.3937 (0.086)	0.4015 (0.251)
3	0.0278 (0.366)	−0.1149 *** (0)	0.527 * (0.031)	0.2915 (0.398)
4	0.0305 (0.311)	−0.0857 *** (0)	0.3457 (0.199)	0.4059 (0.303)
5	−0.0303 (0.223)	−0.1288 *** (0)	0.45 (0.065)	0.1942 (0.548)
6	0.0115 (0.7)	−0.0859 *** (0)	0.2361 (0.363)	−0.3118 (0.391)
7	0.0041 (0.862)	−0.1055 *** (0)	0.3577 (0.126)	0.2314 (0.519)
8	−0.0067 (0.769)	−0.0943 *** (0)	0.5685 * (0.012)	0.2882 (0.412)
9	0.0097 (0.728)	−0.0824 *** (0)	0.2516 (0.268)	−0.0278 (0.932)
10	0.0037(0.89)	−0.0947 ***(0)	0.4027(0.058)	0.1461(0.644)

Note: * *p* < 0.05, *** *p* < 0.001.

**Table A9:** Ten times of the effect’s estimation of air pollution on people’s activity on the randomly sampled on the house or community POIs.

	(i—AQI)	(ii—PM_2.5_)	(iii—AQI)	(iv—PM_2.5_)	(v—AQI)	(vi—PM_2.5_)
Times	Period1	Period1	Period2	Period2	Period3	Period3
1	0.0667 * (0.014)	0.0411 ** (0.002)	0.0742 ** (0.005)	0.2097 *** (0)	0.4271 (0.2)	0.0218 (0.967)
2	0.0643 * (0.014)	0.0435 ** (0.001)	0.0726 ** (0.004)	0.2099 *** (0)	0.0996 (0.737)	−0.8888 * (0.032)
3	0.0694 ** (0.009)	0.0452 *** (0)	0.0453 * (0.034)	0.2119 *** (0)	0.3369 (0.273)	−0.1589 (0.716)
4	0.0748 ** (0.007)	0.0469 *** (0.001)	0.0923 *** (0.001)	0.2553 *** (0)	−0.005 (0.987)	−0.4784 (0.267)
5	0.0675 ** (0.01)	0.0466 *** (0.001)	0.0896 *** (0.001)	0.2431 *** (0)	0.3581 (0.247)	−0.1621 (0.688)
6	0.061 ** (0.008)	0.0352 ** (0.008)	0.0771 ** (0.002)	0.2109 *** (0)	0.4108 (0.255)	−0.6785 (0.143)
7	0.0963 *** (0.001)	0.0489 *** (0.001)	0.0838 *** (0.001)	0.2181 *** (0)	0.6312 * (0.043)	−0.5569 (0.162)
8	0.0743 ** (0.002)	0.0559 *** (0)	0.0853 *** (0.001)	0.2267 *** (0)	0.2482 (0.419)	−0.6419 (0.139)
9	0.0562 * (0.04)	0.039 ** (0.008)	0.0566 * (0.015)	0.1986 *** (0)	0.1134 (0.625)	−0.5035 (0.128)
10	0.071 ** (0.008)	0.0441 *** (0.001)	0.0868 *** (0.001)	0.2655 *** (0)	0.5811 * (0.03)	−0.4768 (0.237)

Note: * *p* < 0.05, ** *p* < 0.01, *** *p* < 0.001.

**Table A10:** Ten times of the effect’s estimation of air pollution on people’s activity on the randomly sampled on the bus stop POIs.

	(i—AQI)	(ii—PM_2.5_)	(iii—AQI)	(iv—PM_2.5_)	(v—AQI)	(vi—PM_2.5_)
Times	Period1	Period1	Period2	Period2	Period3	Period3
1	0.0271 (0.298)	0.0353 ** (0.007)	−0.0231 (0.43)	0.197 *** (0)	0.5775 (0.399)	0.0459 (0.954)
2	0.0319 (0.262)	0.0246 (0.235)	0.0002 (0.997)	0.0996 (0.18)	0.1527 (0.744)	−0.2459 (0.746)
3	0.0405 (0.148)	0.0249 (0.14)	0.0244 (0.564)	0.134 * (0.021)	0.0236 (0.958)	−0.5818 (0.428)
4	0.026 (0.233)	0.0209 (0.229)	−0.0175 (0.648)	0.1333 * (0.013)	0.5704 (0.335)	−0.0521 (0.931)
5	0.0329 (0.195)	0.0378 * (0.018)	−0.011 (0.736)	0.1571 *** (0.001)	−0.3182 (0.518)	−0.6631 (0.348)
6	0.0815 * (0.011)	0.0437 * (0.028)	−0.0069 (0.834)	0.1378 ** (0.01)	−0.3302 (0.579)	−0.6254 (0.446)
7	0.0206 (0.542)	0.0072 (0.752)	0.0054 (0.883)	0.1078 (0.066)	0.033 (0.956)	−0.8677 (0.276)
8	0.0273 (0.316)	0.0299 (0.137)	0.0095 (0.807)	0.161 *** (0.001)	0.0735 (0.925)	−1.2288 (0.199)
9	0.0364 (0.166)	0.0394 * (0.039)	0.0441 (0.17)	0.1548 ** (0.002)	−0.4657 (0.408)	−1.0819 (0.256)
10	0.0406 (0.112)	0.0339 (0.073)	−0.0193 (0.607)	0.0894 (0.171)	0.6506 (0.309)	0.0991 (0.907)

Note: * *p* < 0.05, ** *p* < 0.01, *** *p* < 0.001.

**Table A11:** Ten times of the effect’s estimation of air pollution on people’s activity on the randomly sampled on the subway station POIs.

	(i—AQI)	(ii—PM_2.5_)	(iii—AQI)	(iv—PM_2.5_)	(v—AQI)	(vi—PM_2.5_)
Times	Period1	Period1	Period2	Period2	Period3	Period3
1	0.0399 * (0.046)	0.0117 (0.525)	0.0948 ** (0.001)	0.1623 *** (0)	−0.2442 (0.793)	−1.0232 (0.466)
2	0.0521 * (0.032)	0.0304 (0.076)	0.0376 (0.112)	0.1408 *** (0.001)	−0.7651 (0.109)	−0.5469 (0.444)
3	0.0112 (0.628)	−0.0325 (0.082)	0.0581 * (0.021)	0.1575 *** (0)	0.6758 (0.329)	0.4968 (0.654)
4	0.0439 * (0.041)	0.0321 * (0.042)	0.0367 (0.086)	0.1616 *** (0)	−0.4887 (0.371)	−0.2133 (0.776)
5	0.0651 *** (0.008)	0.0341 (0.057)	0.0159 (0.482)	0.1752 *** (0)	0.2509 (0.65)	−0.872 (0.185)
6	0.0146 (0.469)	−0.0079 (0.652)	0.0307 (0.208)	0.161 *** (0)	0.1355 (0.827)	0.8016 (0.263)
7	0.0479 * (0.022)	−0.0045 (0.824)	0.0491 (0.095)	0.1653 *** (0)	0.3038 (0.671)	0.1252 (0.897)
8	0.0049 (0.825)	0.0094 (0.563)	0.0394 (0.105)	0.2048 *** (0)	0.5727 (0.345)	0.9211 (0.263)
9	0.0342 (0.142)	0.0132 (0.475)	0.0617 * (0.027)	0.18 *** (0)	−0.6704 (0.354)	−1.2534 (0.162)
10	0.0431 (0.061)	0.0177 (0.298)	0.0746 * (0.011)	0.1702 *** (0)	−0.0914 (0.881)	0.2375 (0.722)

Note: * *p* < 0.05, ** *p* < 0.01, *** *p* < 0.001.

**Table A12:** Estimation of the effects of air pollution on people’s activity on the sightseeing POI.

	(i—AQI)	(ii—PM_2.5_)	(iii—AQI)	(iv—PM_2.5_)	(v—AQI)	(vi—PM_2.5_)
**Dependent variables**	Period1	Period1	Period2	Period2	Period3	Period3
AQI	0.0570 ***		0.00195		0.0744	
	(0.0145)		(0.0242)		(0.1343)	
PM_2.5_		0.00949		−0.00784		−0.794 ***
		(0.0093)		(0.0269)		(0.1658)
**Weather variables**						
TEMP	2.264	2.446	206.5 ***	201.9 ***	100.5 **	−19.3
	(2.3320)	(2.1031)	(18.3392)	(23.3036)	(31.1725)	(20.4207)
(TEMP)^2^	−0.0769 **	−0.0754 **	−2.701 ***	−2.642 ***	−2.227 ***	0.19
	(0.0254)	(0.0229)	(0.2376)	(0.2982)	(0.6226)	(0.4093)
WIND	2.534 ***	2.790 ***	15.58 ***	15.56 ***	54.01 ***	15.93 *
	(0.2617)	(0.2194)	(0.9970)	(0.9738)	(5.7752)	(7.4903)
CLOUD	7.730 ***	8.616 ***	−21.49 ***	−21.31 ***	0	0
	(0.8331)	(0.7992)	(1.5167)	(1.3588)	−	−
Constant	402.6 ***	398.0 ***	−3436 ***	−3346 ***	−632.4	1088.0 ***
	(52.2414)	(47.5121)	(351.9851)	(450.4228)	(416.5775)	(298.0420)
POI fixed effects	YES	YES	YES	YES	YES	YES
Type of day fixed effects	YES	YES	YES	YES	YES	YES
N	4000	4000	4000	4000	2200	2200
R^2^	0.4525	0.452	0.277	0.277	0.3775	0.3795

Note: The dependent variable is the mobile phone users’ density (MPUD) on a POI in a period. Robust standard errors are clustered by POI and reported in parentheses; ** *p* < 0.01, *** *p* < 0.001.

**Table A13:** Estimation of the effects of air pollution on the activity of eating out in restaurant POI.

	(i—AQI)	(ii—PM_2.5_)	(iii—AQI)	(iv—PM_2.5_)
**Dependent variables**	Lunch	Lunch	Dinner	Dinner
AQI	0.00374		0.620 ***	
	(0.0198)		(0.1661)	
PM_2.5_		−0.0994 ***		0.391
		(0.0144)		(0.2482)
**Weather variables**				
TEMP	−376.0 ***	−378.7 ***	−244.8 ***	−210.0 ***
	(15.8275)	(16.0107)	(17.3950)	(29.7944)
(TEMP)^2^	4.711 ***	4.748 ***	6.258 ***	5.588 ***
	(0.1979)	(0.1999)	(0.4188)	(0.6805)
WIND	23.60 ***	23.57 ***	93.60 ***	89.30 ***
	(1.4692)	(1.4384)	(5.6235)	(5.6046)
CLOUD	−41.08 ***	−38.94 ***	0	0
	(2.8149)	(2.6028)	−	−
Constant	7867.2 ***	7926.1 ***	2564.5 ***	2177.8 ***
	(301.4089)	(306.0135)	(156.5763)	(341.0804)
POI fixed effects	YES	YES	YES	YES
Type of day fixed effects	YES	YES	YES	YES
N	3780	3780	2268	2268
R^2^	0.5421	0.5425	0.6658	0.6655

Note: The dependent variable is the mobile phone users’ density (MPUD) on a POI in a period. Robust standard errors are clustered by POI and reported in parentheses; *** *p* < 0.001.

**Table A14:** Estimation of the effect of air pollution on people’s staying-in activity for the house/community POI.

	(i—AQI)	(ii—PM_2.5_)	(iii—AQI)	(iv—PM_2.5_)	(v—AQI)	(vi—PM_2.5_)
**Dependent variables**	Period1	Period1	Period2	Period2	Period3	Period3
AQI	0.0614 ***		0.0704 ***		0.375	
	(0.0175)		(0.0171)		(0.2090)	
PM_2.5_		0.0393 ***		0.225 ***		−0.403
		(0.0091)		(0.0144)		(0.2895)
**Weather variables**						
TEMP	14.22 ***	13.35 ***	342.6 ***	451.4 ***	240.5 ***	114.0 **
	(1.7454)	(1.6242)	(29.4455)	(34.5974)	(44.3278)	(37.9603)
(TEMP)^2^	−0.217 ***	−0.206 ***	−4.424 ***	−5.799 ***	−5.067 ***	−2.511 **
	(0.0192)	(0.0179)	(0.3826)	(0.4478)	(0.8937)	(0.7672)
WIND	2.656 ***	2.996 ***	19.01 ***	19.78 ***	93.94 ***	66.43 ***
	(0.2548)	(0.2106)	(1.3030)	(1.3243)	(8.8098)	(15.2918)
CLOUD	12.40 ***	12.00 ***	−18.56 ***	−22.73 ***	0	0
	(1.0094)	(1.0000)	(1.3265)	(1.3514)	−	−
Constant	279.9 ***	296.8 ***	−5923 ***	−8050 ***	−2277.7 ***	−528.7
	(40.8030)	(38.2207)	(562.706)	(664.212)	(591.1757)	(549.7147)
POI fixed effects	YES	YES	YES	YES	YES	YES
Type of day fixed effects	YES	YES	YES	YES	YES	YES
N	4000	4000	4000	4000	2200	2200
R^2^	0.5943	0.5943	0.4793	0.4814	0.5618	0.5618

Note: The dependent variable is the mobile phone users’ density (MPUD) on a POI in a period. Robust standard errors are clustered by POI and reported in parentheses; ** *p* < 0.01, *** *p* < 0.001.

**Table A15:** Estimations of effects of air pollution on people’s activity of transportation mode options on bus stop POI.

	(i—AQI)	(ii—PM_2.5_)	(iii—AQI)	(iv—PM_2.5_)	(v—AQI)	(vi—PM_2.5_)
**Dependent variables**	Period1	Period1	Period2	Period2	Period3	Period3
AQI	0.036		0.0142		0.17	
	(0.0193)		(0.0263)		(0.3953)	
PM_2.5_		0.0217		0.147 ***		−0.342
		(0.0136)		(0.0383)		(0.5427)
**Weather variables**						
TEMP	5.526	5.049	296.6 ***	373.7 ***	190.8 *	112
	(3.9977)	(3.6908)	(31.9462)	(38.7465)	(82.4009)	(68.1898)
(TEMP)^2^	−0.129 **	−0.123 **	−3.832 ***	−4.808 ***	−4.059 *	−2.469
	(0.0433)	(0.0402)	(0.4170)	(0.4995)	(1.6581)	(1.3709)
WIND	3.668 ***	3.860 ***	18.86 ***	19.31 ***	83.43 ***	63.88 **
	(0.4218)	(0.3523)	(1.6356)	(1.6282)	(12.6869)	(24.2800)
CLOUD	12.28 ***	12.11 ***	−22.85 ***	−25.77 ***	0	0
	(1.2568)	(1.0827)	(2.1693)	(1.9706)	−	−
Constant	522.5 ***	532.0 ***	−5028.9 ***	−6535.0 ***	−1620.6	−517.1
	(88.3302)	(81.8273)	(609.2183)	(745.6948)	(1100)	(996.0337)
POI fixed effects	YES	YES	YES	YES	YES	YES
Type of day fixed effects	YES	YES	YES	YES	YES	YES
N	1980	1980	1980	1980	1089	1089
R^2^	0.5687	0.5686	0.4249	0.426	0.5603	0.5603

Note: The dependent variable is the mobile phone users’ density (MPUD) on a POI, in a period. Robust standard errors are clustered by POI and reported in parentheses; * *p* < 0.05, ** *p* < 0.01, *** *p* < 0.001.

**Table A16:** Estimations of the effects of air pollution on people’s transport mode subway station POI.

	(i—AQI)	(ii—PM_2.5_)	(iii—AQI)	(iv—PM_2.5_)	(v—AQI)	(vi—PM_2.5_)
**Dependent variables**	Period1	Period1	Period2	Period2	Period3	Period3
AQI	0.031		0.0567 **		−0.00762	
	(0.0159)		(0.0184)		(0.4551)	
PM_2.5_		0.00557		0.167 ***		−0.18
		(0.0127)		(0.0272)		(0.6530)
**Weather variables**						
TEMP	10.44 **	10.53 **	333.8 ***	411.8 ***	183.4	161.1
	(3.3324)	(3.1121)	(42.0320)	(48.6495)	(93.7985)	(81.4032)
(TEMP)^2^	−0.186 ***	−0.185 ***	−4.306 ***	−5.291 ***	−3.935 *	−3.486 *
	(0.0346)	(0.0325)	(0.5481)	(0.6306)	(1.8950)	(1.6519)
WIND	3.938 ***	4.072 ***	20.71 ***	21.30 ***	89.90 ***	82.09 **
	(0.3957)	(0.3453)	(1.9061)	(1.9226)	(14.7067)	(30.4025)
CLOUD	13.99 ***	14.47 ***	−26.44 ***	−29.32 ***	0	0
	(1.2415)	(1.1751)	(2.7577)	(2.5480)	−	−
Constant	472.7 ***	470.3 ***	−5656.0 ***	−7181.8 ***	−1374.6	−1049.3
	(78.7130)	(73.8032)	(800.5169)	(931.2908)	(1200)	(1200)
POI fixed effects	YES	YES	YES	YES	YES	YES
Type of day fixed effects	YES	YES	YES	YES	YES	YES
N	1960	1960	1960	1960	1078	1078
R^2^	0.5725	0.5725	0.4474	0.4482	0.55	0.55

Note: The dependent variable is the mobile phone users’ density (MPUD on a POI in a period. Robust standard errors are clustered by POI and reported in parentheses; * *p* < 0.05, ** *p* < 0.01, *** *p* < 0.001.

### Appendix C.3. The Results of the Mutilative Linear Regression

We input the time series into the regression model given in Equation (14) in the main manuscript. The Table A17 and Table A18 are the results for the groups’ bike riding people and walking people. For each table, columns (i) and (ii) show the estimation of the number of people in the group impacted by AQI and PM_2.5_, while the other two columns show the distance. Because the input data are time series, they might have an autocorrelation. Hence use Prais-Winsten (PW) method to decrease the negative impact of this. The comparison between the Durbin-Watson test value before and after using the PW method are shown in Table A19, which shows the benefits of using the PW method to decrease the negative impact of autocorrelation.

**Table A17:** Estimations of the effects of air pollution on people bike riding.

	(i—AQI)	(ii—PM_2.5_)	(iii—AQI)	(iv—PM_2.5_)
	Number	Number	Distance	Distance
AQI	−19.54 *		−7.271 *	
	(8.6284)		(3.5220)	
PM_2.5_		−2.839		−1.015
		(6.5185)		(2.7622)
TEMP	255.9	−37.73	116.7	−3.994
	(277.4387)	(292.0201)	(123.4601)	(128.0484)
(TEMP)^2^	−6.352	−2.794	−2.831	−1.403
	(3.5965)	(3.8598)	(1.6012)	(1.6949)
WIND	124.4	95.25	56.04 *	44.06
	(62.5282)	(63.9672)	(27.6811)	(28.0982)
CLOUD	−1147.5 **	−830.4	−497.8 *	−360.2
	(425.6531)	(505.6191)	(192.2372)	(226.7534)
Constant	7563.8	9745.2	2855.4	3988
	(5700)	(5700)	(2500)	(2500)
Type of day controls	YES	YES	YES	YES
Hour controls	YES	YES	YES	YES
N	73	73	73	73
R^2^	0.3971	0.3983	0.3838	0.3989

Note: The dependent variable is the number of people who ride a general bike, or their distance moved hourly in Beijing. Standard errors are reported in parentheses; * *p* < 0.05, ** *p* < 0.01.

**Table A18:** Estimations of the effects of air pollution on people walking.

	(i—AQI)	(ii—PM_2.5_)	(iii—AQI)	(iv—PM_2.5_)
	Number	Number	Distance	Distance
AQI	−7.466 *		−1.201 *	
	(3.3690)		(0.5445)	
PM_2.5_		−0.661		−0.207
		(2.3764)		(0.4006)
TEMP	30.77	−35.41	7.162	−3.113
	(84.8118)	(90.3629)	(14.7112)	(15.6714)
(TEMP)^2^	−1.202	−0.343	−0.23	−0.103
	(1.1018)	(1.1933)	(0.1909)	(0.2069)
WIND	25.21	22.78	5.529	5.127
	(19.4659)	(20.3324)	(3.3585)	(3.5008)
CLOUD	−360.4 **	−293.3	−59.49 **	−44.84
	(126.2527)	(151.0873)	(22.0692)	(26.2204)
Constant	3506.3	3132.2	542.1	506.2
	(1800)	(1800)	(309.7733)	(316.8005)
Type of day controls	YES	YES	YES	YES
Hour controls	YES	YES	YES	YES
N	73	73	73	73
R^2^	0.4087	0.3661	0.404	0.3682

Note: The dependent variable is the number of people who walk, or their distance moved hourly in Beijing. Standard errors are reported in parentheses; * *p* < 0.05, ** *p* < 0.01.

**Table A19:** Comparison between the Durbin-Watson statistic before and after using the PW method.

	Number of People		Distance of Movement	
	AQI	PM_2.5_	AQI	PM_2.5_
Walk	1.818	1.778	1.889	1.818
	(0.423)	(0.394)	(0.415)	(0.391)
Riding bike	1.929	1.856	1.893	1.838
	(0.404)	(0.376)	(0.397)	(0.372)

Note: the values of the transformed Durbin-Watson statistic after the Prais-Winsten estimation are shown, while the original Durbin-Watson statistic is in parentheses; The closer the value is to 2, the smaller the autocorrelation sequence, and vice versa.
